# Region-specific reversal of epidermal planar polarity in the *rosette* fancy mouse

**DOI:** 10.1242/dev.202078

**Published:** 2023-09-07

**Authors:** Maureen Cetera, Rishabh Sharan, Gabriela Hayward-Lara, Brooke Phillips, Abhishek Biswas, Madalene Halley, Evalyn Beall, Bridgett vonHoldt, Danelle Devenport

**Affiliations:** ^1^ Department of Genetics, Cell Biology and Development, University of Minnesota Twin Cities, Minneapolis, MN 55455, USA; ^2^ Lewis-Sigler Institute for Integrative Genomics, Princeton University, Princeton, NJ 08540, USA; ^3^ Department of Molecular Biology, Princeton University, Princeton, NJ 08540, USA; ^4^ Research Computing, Office of Information Technology, Princeton University, Princeton, NJ 08540, USA; ^5^ Department of Ecology and Evolutionary Biology, Princeton University, Princeton, NJ 08540, USA

**Keywords:** Planar cell polarity, Frizzled 6, Hair follicle, Epidermis, Morphogenesis, Mouse

## Abstract

The planar cell polarity (PCP) pathway collectively orients cells with respect to a body axis. Hair follicles of the murine epidermis provide a striking readout of PCP activity in their uniform alignment across the skin. Here, we characterize, from the molecular to tissue-scale, PCP establishment in the *rosette* fancy mouse, a natural variant with posterior-specific whorls in its fur, to understand how epidermal polarity is coordinated across the tissue. We find that *rosette* hair follicles emerge with reversed orientations specifically in the posterior region, creating a mirror image of epidermal polarity. The *rosette* trait is associated with a missense mutation in the core PCP gene *Fzd6*, which alters a consensus site for N-linked glycosylation, inhibiting its membrane localization. Unexpectedly, the Fzd6 trafficking defect does not block asymmetric localization of the other PCP proteins. Rather, the normally uniform axis of PCP asymmetry rotates where the PCP-directed cell movements that orient follicles are reversed, suggesting the PCP axis rotates 180°. Collectively, our multiscale analysis of epidermal polarity reveals PCP patterning can be regionally decoupled to produce posterior whorls in the *rosette* fancy mouse.

## Teaser

**Highlighted Article:** Region-specific rotation of the planar cell polarity axis correlates with reversed hair follicle orientation in the *rosette* fancy mouse.

## INTRODUCTION

Epidermal appendages such as hair, feathers, scales and bristles are uniformly aligned along the body of an animal. This tissue-level pattern is a striking example of planar cell polarity (PCP), in which thousands of cells collectively orient in a common direction (Devenport and Fuchs, 2008). The remarkable scale of this coordination is achieved through cell-to-cell communication mediated by a set of highly conserved membrane-associated proteins collectively known as the core PCP pathway. Through a process that is not well understood, core PCP proteins ‘read’ long-range directional cues to orient cell polarity relative to the body axes (Fisher and Strutt, 2019). The PCP machinery functions in a vast array of diverse cell types and tissues to drive collective behaviors including neural tube closure, organization of stereocilia bundles and directional cilia beating (Butler and Wallingford, 2017; Davey and Moens, 2017). Therefore, deciphering mechanisms controlling PCP in highly accessible tissues such as the surface ectoderm can yield fundamental insights into PCP function in other less accessible but vital processes that shape tissues and organs during embryonic development. A hallmark of PCP organization is its long-range coordination with a body or tissue axis. Despite this common feature, the mechanisms that link the axis of PCP asymmetry to a body axis remain elusive.

The core components of the PCP pathway were discovered using forward mutagenesis screens in *Drosophila,* where misoriented wing hairs and cuticular bristles served as phenotypic indicators of defective planar polarity. The PCP genes *frizzled*, *disheveled*, *prickle*, *starry night* (also known as *flamingo*) and *Van Gogh* were aptly named based on their mutant phenotypes of disordered or swirled hair and bristle patterns (Wolff and Rubin, 1998; Taylor et al., 1998; Gubb and García-Bellido, 1982; Krasnow et al., 1995). In other species where mutagenesis is less practical, natural genetic variation can provide a powerful means to uncover new genes or novel mutations in developmental pathways in an unbiased way. For example, animal breeders have selected for spontaneous mutations in birds and rodents that give rise to beautiful feather and hair patterns, such as the collar of the crested pigeon or the whorls of the Abyssinian guinea pig (Shapiro et al., 2013; Castle, 1905). In both cases, the polarity of epidermal appendages no longer uniformly aligns with the anterior-posterior (AP) body axis, suggesting tissue-level planar polarity is disrupted. PCP is an ideal developmental pathway to genetically dissect using natural variation because misaligned epidermal structures are easily visualized by the naked eye, producing traits that are desirable to breeders. Identifying the sequence variants associated with unique epidermal patterns will likely uncover previously unreported mutations in the PCP pathway itself or new genes required for PCP patterning.

**Fig. 1.**
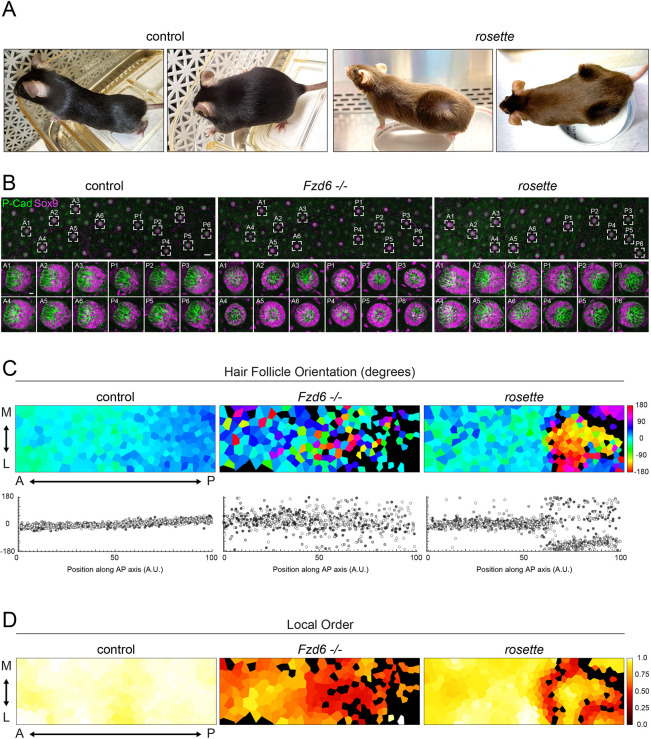
**Posterior hair follicles emerge with reversed orientations in *rosette* mutants.** (A) Hair patterns of C57BL/6 controls (left) and the *rosette* fancy mouse (right). (B-D) Orientation of emerging hair follicles at E15.5. (B) Representative scanning confocal projections of whole-mount dorsal skins labeled with the asymmetrically distributed follicle markers P-Cad (green) and Sox9 (magenta). Scale bar: 100 μm. Zoomed in view of individual follicles are shown below. A1, A2, etc. indicate anterior follicles; P1, P2, etc. indicate posterior follicles. Scale bar: 10 μm. (C, top) Voronoi diagrams of follicle orientation represented by color (cool colors, anteriorly oriented; warm colors, posteriorly oriented; black, unpolarized). A↔P, anterior-posterior; M↔L, medial-lateral. (C, bottom) Graph showing individual follicle orientation along the AP axis. The three shades indicate three embryos per genotype. (D) Voronoi diagrams representing local order of follicle orientations (high coordination, white; low coordination, red; unpolarized, black).

The ‘rosette’ trait in the mouse, *Mus musculus*, is a unique coat pattern where the dorsal fur forms two large symmetrical whorls, or rosettes, in the posterior half of the body (Fig. 1A). The phenotype appeared in a British fancier's colony in the 1960s and is now a coveted trait in the breeding community (King, 1975; Wallace, 1971). The recessive phenotype can be combined with a number of different coat colors, textures and lengths to create truly unique animals. The *rosette* trait shares features with the hair phenotypes caused by mutations in PCP pathway genes in laboratory mice, but the region-specific nature of the coat pattern sets it apart from previously described PCP mutants. Of the PCP mutant strains that survive to adulthood, hair whorls typically arise over the entire skin surface (Ravni et al., 2009; Guo et al., 2004). Furthermore, loss-of-function mutations in PCP genes tend to cause neural tube defects and embryonic lethality, thus skin-specific knockouts are required to observe hair patterning defects (Cetera et al., 2017; Curtin et al., 2003; Murdoch et al., 2001; Kibar et al., 2001). These key differences suggest *rosette* is a novel variant that alters PCP signaling in a region-specific manner.

Here, we describe how the extraordinary *rosette* coat pattern emerges during skin development at the cellular, appendage and tissue-wide scales, and show that the whorls are the result of aberrant PCP establishment. We find that posterior whorls develop from hair follicles that emerge with reversed orientations during embryonic stages – the time at which the PCP pathway initially acts to polarize nascent hair follicles. Genetic mapping revealed that mice displaying the *rosette* trait are homozygous for a previously unreported missense mutation in the *Fzd6* locus, a known core component of the PCP pathway. We show that the missense mutation, which is predicted to inhibit N-linked glycosylation of the Fzd6 extracellular domain, interferes with proper Fzd6 trafficking through the secretory system and prevents its delivery to the plasma membrane. Under this condition, Fzd6 accumulates intracellularly in the skin epithelium, whereas the other core PCP components become polarized but are not uniformly coordinated across the tissue. In the posterior, the PCP axis loses its tissue-level coordination and rotates where the PCP-directed cell movements that polarize the follicle are reversed, suggesting a full 180° rotation of the PCP axis. Collectively, our analysis of epidermal polarity in *rosette* embryos reveals Fzd6 plays a role in directing the orientation of PCP in the posterior region of the skin and suggests that different skin regions can independently establish an axis of PCP asymmetry.

## RESULTS

### Posterior hair follicles emerge with reversed orientations in *rosette* mutants

The ‘*rosette*’ fancy mouse is prized for its distinctive coat marked by posterior-specific whorls (Fig. 1A). Despite gaining popularity among fanciers, *rosette* mice were not available to the scientific community. This led us to attend regional fancy mouse shows where we networked with the breeding community to obtain and rederive the whorled mice in a laboratory setting. Although swirling hair patterns in mice are indicative of a defect in the PCP pathway, the regionality of the *rosette* phenotype is highly unusual, as PCP is required to align hair follicles across the entire skin (Ravni et al., 2009; Cetera et al., 2017; Guo et al., 2004). Hair follicle polarity is established during embryonic development shortly after hair placodes are specified from the basal progenitor layer of the embryonic epidermis (Devenport and Fuchs, 2008). Asymmetrically localized PCP proteins direct polarized cell movements within each placode, causing the multicellular structure to tilt and grow in an anterior orientation (Cetera et al., 2018). In the absence of PCP function, hair placodes emerge with randomized orientations or they fail to polarize altogether and grow vertically into the dermis (Devenport and Fuchs, 2008; Cetera et al., 2017; Wang et al., 2010; Dong et al., 2018; Basta et al., 2023). To determine whether the *rosette* phenotype arises during the initial stages of hair follicle polarization, we quantified hair placode orientations in *rosette* mutant embryos at E15.5 and compared these with wild-type controls as well as a loss-of-function PCP mutant (*Fzd6^−/−^*).

The orientation of embryonic follicles can be determined by examining the relative positions of P-Cadherin- (P-Cad) and Sox9-expressing cells within individual follicles. In wild-type embryos, these two cell populations are asymmetrically positioned at the anterior and posterior sides of each follicle, with P-Cad-expressing cells oriented toward the head of the animal (Fig. 1B, panels A1-6 and P1-6, Fig. S1A). By contrast, in *Fz6^−/−^* mutant follicles, P-Cad- and Sox9-expressing cells are either mispositioned relative to the AP axis (Fig. 1B, panels A1, A2, A3 and P6) or unpolarized (Fig. 1B, panels A4, A5 and P1-5) with Sox9-expressing cells encircling a central cluster of P-Cad-expressing cells (Cetera et al., 2017; Wang et al., 2010). The embryonic hair pattern of *rosette* mutants, by contrast, is distinct. Follicles located in the anterior half of the embryo position P-Cad-expressing cells anteriorly (A1-6), as they do in wild-type controls, whereas follicles in the posterior region of the embryo are reversed, with P-Cad- and Sox9-expressing populations inverted relative to the AP axis (Fig. 1B, panels P1-6).

To characterize tissue-scale hair follicle patterns in each genotype, we used a semi-automated method to measure follicle orientations based on P-Cad and Sox9 distributions (Basta et al., 2023). We displayed this information at the tissue level in Voronoi diagrams where each polygon represents a follicle that is color-coded according to its orientation from 0° to 360° (Fig. 1C, top; cool colors, anteriorly oriented; warm colors, posteriorly oriented; black, unpolarized, Fig. S1A). To determine reproducibility between individuals, follicle orientations were plotted relative to their AP positions across multiple embryos (Fig. 1C, bottom). In control embryos, follicles point anteriorly and are well aligned between +45° and −45°. Follicle orientations in *Fzd6^−/−^* mutants are much more broadly distributed, ranging from +180° to −180°, with unpolarized follicles scattered across the tissue. In *rosette* embryos, the majority of follicles correctly align between +45° and −45° in the anterior half of the skin, whereas follicles in the posterior region are reversed, predominantly aligning near +180° and −180°.

As a third metric to describe the collectivity of follicle orientations, we defined an order parameter that compares the relative angles of neighboring follicles (Fig. 1D, Eqn 1 in the Materials and Methods). Parallel hair follicles are highly ordered with an order parameter of one (Fig. 1D, white), whereas antiparallel hair follicles have an order parameter of zero (dark red). In control embryos, local order is high across the entire skin (Fig. 1D, white and yellow), whereas in *Fzd6^−/−^* mutants, local order is low, with each follicle appearing to orient independently of its neighbors (Fig. 1D, orange and red). By contrast, local order is high in both the anterior and posterior regions of *rosette* embryos (Fig. 1D, yellow) with the exception of a sharp zone of low order surrounding the reversed patch of posterior follicles. In this region, follicles are changing their orientation or growing straight down (Fig. 1D, black), resulting in low coordination between neighboring follicles (Fig. 1D, red). Together, these data demonstrate that hair follicle polarity is altered in a region-specific manner in *rosette* mutants, and these defects arise during the earliest stages of hair follicle polarization. Moreover, the reversed orientations and high local order of follicles in *rosette* mutants comprise a phenotype that is distinct from a loss-of-function PCP mutant.

### Coordinated hair follicle reversal persists into postnatal stages

**Fig. 2.**
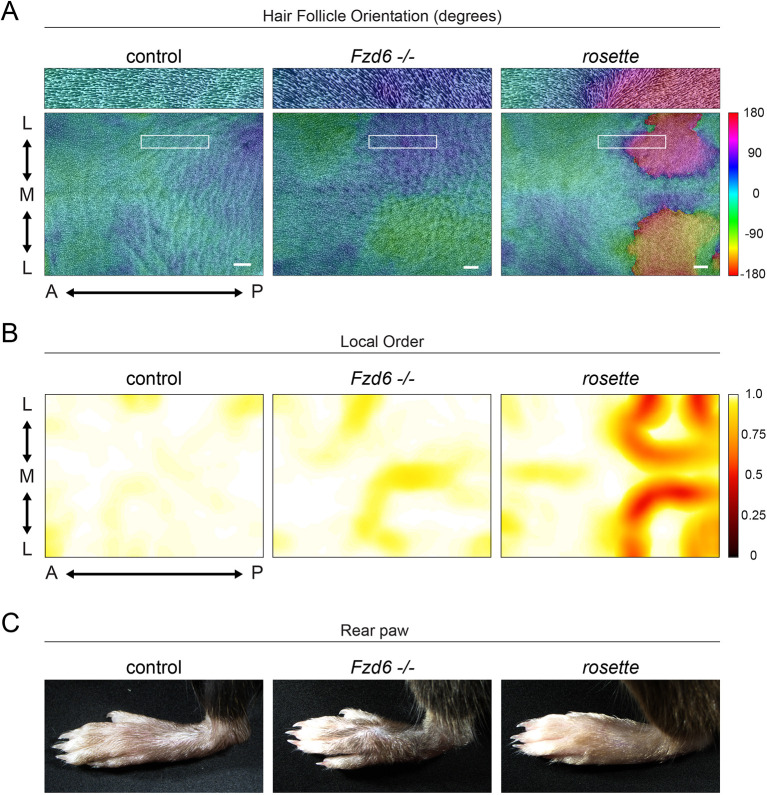
**Coordinated hair follicle reversal persists into postnatal stages.** (A) Representative images of cleared, flat-mounted dorsal skins at P4 from *rosette/+* controls (left), *Fzd6^−/−^* (center) and *rosette* (right). Hair follicle orientation is indicated by color (cool colors, anteriorly oriented; warm colors, posteriorly oriented). Scale bars: 1 mm. (B) Local order of follicle orientation (high order, white; low order, red). A↔P, anterior-posterior. M↔L, medial-lateral, with M indicating the midline. (C) Rear paws from indicated genotypes.

Misoriented hair follicles in PCP mutants are not static but instead have the remarkable ability to rotate up to 180°. These rotations minimize the angular difference between adjacent hair follicles during postnatal stages, which ultimately gives rise to the elaborate whorls, ridges and crosses that mark the coats of specific PCP mutants (Wang et al., 2010, 2006; Cetera et al., 2017). Postnatal follicle rotations can also correct an initially disordered follicle pattern. For example, in *Fzd6^−/−^* mutants, initially misoriented hairs rotate and correct their alignment along the AP axis, and, by postnatal day 7 (P7), their coats are indistinguishable from wild-type animals mutants (Cetera et al., 2017). To examine how the *rosette* hair pattern evolves and refines during postnatal stages, we measured hair follicle angles and local order across the entire dorsal skin at postnatal day 4 (P4) when *Fzd6^−/−^* follicles are in the process of reorienting. At this stage in *Fzd6^−/−^* mutants, most follicles have rotated to face anteriorly or point slightly away from the midline, particularly in the posterior region, as previously described (Fig. 2A, Fig. S2, cool colors, Fig. S1B) (Cetera et al., 2017). Furthermore, previously uncoordinated follicles at embryonic stages are highly ordered by P4 (Fig. 2B, Fig. S2, white/yellow). In contrast, follicles in the posterior region of *rosette* mutants retained their reversed, posterior-facing orientations, even at P4, when follicles have the ability to rotate into correct orientations (Fig. 2A, Fig. S2, warm colors). The zones of high and low local order that were observed in embryonic stages were also maintained at P4. Interestingly, all previously reported PCP mutants display rear paw whorls that are maintained throughout postnatal stages, but they are not present in *rosette* animals (Fig. 2C) (Cetera et al., 2017; Guo et al., 2004; Basta et al., 2023; Ravni et al., 2009). These data show that misoriented follicles in the *rosette* mutant fail to correct their orientation during early postnatal stages and *rosette* mutants lack the characteristic PCP-mutant paw whorls.

### The *rosette* phenotype correlates with a missense mutation in *Fzd6*

**Fig. 3.**
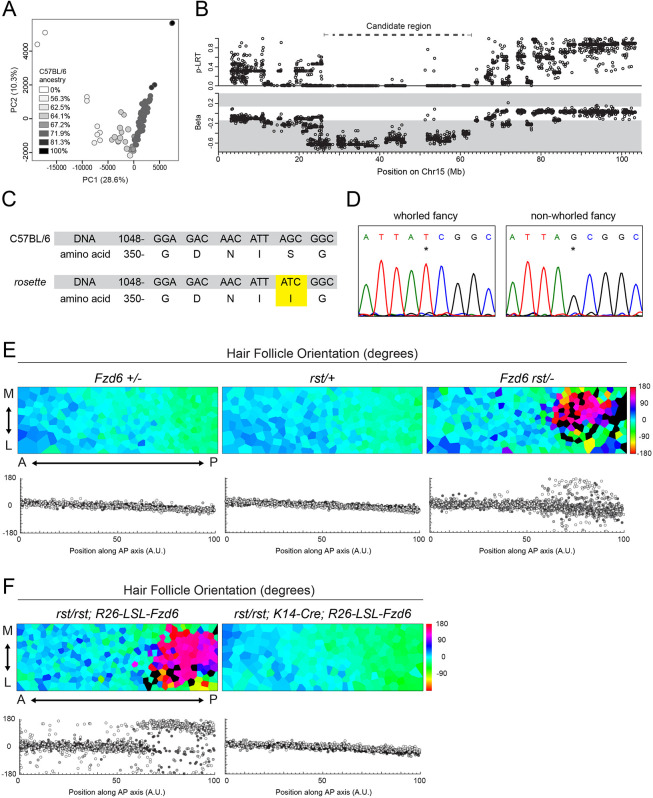
**The *rosette* phenotype correlates with a missense mutation in *Fzd6*.** (A) PCA of the filtered SNP dataset for all mice analyzed. Circle shading indicates C57BL/6 ancestry. Percent variation for the first two axes are included. (B) Candidate region associated with the whorled phenotype on chromosome 15 (dashed line) identified by outlier loci from the likelihood ratio test statistic (top panel) and outlier β values (lower panel, gray shading). (C) A novel missense mutation in the *rosette* mutant compared with the C57BL/6 reference sequence. G1061>A results in an amino acid change in the Fzd6 protein, S354I (yellow). (D) Sanger sequencing of Fzd6 position 1061 (asterisk) from *rosette* and non-whorled fancy mice outside our colony. (E,F) (Top) Voronoi diagrams of hair follicle orientations at E15.5 (cool colors, anteriorly oriented; warm colors, posteriorly oriented; black, unpolarized). A↔P, anterior-posterior; M↔L, medial-lateral. (Bottom) Graphs showing individual follicle orientations with respect to AP position. Three different shades indicate three embryos per genotype.

To uncover genetic elements associated with the *rosette* phenotype, we leveraged the diversity between the genetic backgrounds of fancy and laboratory mice. Although common lab strains originated from fancy mice, they are derived from a limited number of founders and are highly inbred (Phifer-Rixey and Nachman, 2015). The background of fancy mice, however, is mixed, and animals are derived from multiple sources to obtain the unique combination of traits desired by breeders. The phenotypic animals in our colony are descendants of a single *rosette* founder crossed to the C57BL/6 inbred lab strain. Using a Mouse Diversity Genotyping Array, we identified SNPs in the fancy background that co-segregate with the appearance of posterior whorls (Yang et al., 2009). We analyzed 91 animals in total, including the founder, its *rosette* sibling, two C57BL/6 animals and descendants from the fancy founder with increasing levels of C57BL/6 ancestry (Tables S1 and S2). After applying a minor allele frequency of <0.3%, we retained 67,589 SNP loci that capture the gradient of C57BL/6 ancestry in principle component analysis 1, showing that the maximum variance is dependent on the genetic background (Fig. 3A). We used a linear mixed model to identify SNP variants associated with the whorl phenotype and used the likelihood ratio test (LRT) for significance testing (Zhou and Stephens, 2012). We prioritized SNPs with allelic effect (β) values in the lower or upper 5th percentile of the distribution, and considered these putative outlier SNPs. By integrating the LRT *P*-value and β values, we identified a candidate region on chromosome 15 that contained 132 genes, including *Fzd6* (Fig. 3B). Despite the distinct hair patterning defects displayed in *Fzd6^−/−^* and *rosette* mutants, we reasoned that a previously unreported mutation in *Fzd6* might contribute to the unique *rosette* phenotype.

Exon sequencing of the *Fzd6* gene in phenotypic *rosette* animals identified four point mutations not present in the C57BL/6 reference genome, three of which are known SNP variants found between lab strains (Table S3) (Lilue et al., 2018). The fourth is a novel missense mutation, G1061>A, that results in a serine-to-isoleucine substitution at amino acid 354 (S354I, Fig. 3C). To determine whether the missense mutation was linked to the *rosette* phenotype in mice outside our colony, we sequenced the same region in three unrelated *rosette* animals obtained from an independent breeder. All three animals had the G1061>A mutation in *Fzd6* (Fig. 3D). To rule out the possibility that G1061>A arose in the background of fancy animals independently of their whorled status, three animals with a normal coat pattern from the breeder's colony were sequenced. None carried the mutation, revealing the G1061>A mutation in Fzd6 correlates with the *rosette* phenotype (Fig. 3D). To test for a genetic interaction between *rosette* and *Fzd6*, phenotypic *rosette* animals were crossed to *Fzd6^−/−^* homozygotes and their heterozygous progeny were scored for hair polarity defects. If the S354I substitution impairs Fzd6 function, it should fail to complement the *Fzd6* null allele (Fig. 3E, Fig. S1A). We found that although hair patterning is normal in embryos heterozygous for *Fzd6^+/−^* or *rosette/+* alone, when they are present in combination, follicles in the posterior region are misoriented, indicating the S354I mutation likely alters Fzd6 function.

Interestingly, the S354I allele shows a stronger phenotype in homozygous animals than when heterozygous with the null allele (Fig. 1C, compared with Fig. 3E). If the S354I substitution is a hypomorph, we would expect a stronger, not weaker, phenotype in combination with the null allele. One potential explanation is that a recessive modifier is present in the *rosette* background that is missing in our *Fz6* knockout lab strain. A previous study showed that the *Fzd6^−/−^* follicle pattern can be enhanced when combined with a naturally occurring deletion of exon 5 in astrotactin 2 (*Astn2*) found in the129X1/SvJ lab strain (Chang et al., 2015b). However, we did not detect the deletion in the fancy *rosette* background (Fig. S3). Although this result does not rule out the possibility that another modifier could contribute to the phenotype, it does show reversal cannot be explained by combining previously described alleles. If impaired Fzd6 function underlies the coat patterning phenotype of *rosette* mutants, expressing an exogenous copy of wild-type *Fzd6* should rescue the hair follicle reversals. We therefore overexpressed Fzd6 in *rosette* mutants by driving expression of the *Rosa26 lox-stop-lox Fzd6WT* transgene with a skin-specific driver, *K14-Cre* (Hua et al., 2014; Vasioukhin et al., 1999) (Fig. 3F, Fig. S1A). In the absence of *K14-Cre*, the polarity of *rosette* mutant hair follicles was unchanged; follicles in the posterior region were reversed. However, upon expression of wild-type *Fzd6* with *K14-Cre*, all follicles were collectively oriented toward the anterior of the animal, restoring the wild-type pattern. Together, data from our mapping, genetic interaction and rescue experiments strongly suggest that altered Fzd6 function contributes to the *rosette* phenotype.

### Membrane localization of Fz6 is lost in *rosette* mutants

**Fig. 4.**
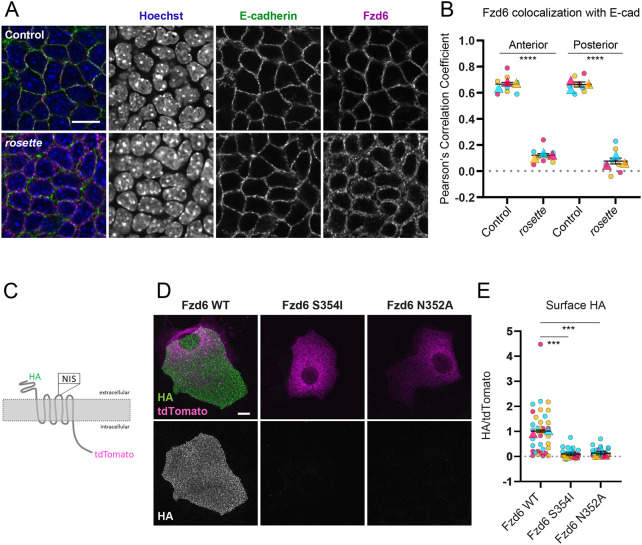
**Membrane localization of Fz6 is lost in *rosette* mutants.** (A) Scanning confocal images of whole-mount epidermis labeled with E-cadherin and Fzd6 antibodies and Hoechst in control (top) and *rosette* (bottom) embryos at E15.5. (B) Colocalization of E-cadherin and Fzd6 represented as Pearson's correlation coefficient. Three animals were analyzed per genotype, represented by unique colors. Circles indicate a single analyzed image; the triangle is the average/animal. Data are mean±s.e.m. across all animals per group. Two-tailed *t*-test: *P*<0.00001, anterior; *P*<0.00005, posterior. (C) Schematic of tagged Fzd6 constructs. (D) Keratinocytes transfected with HA-Fzd6-tdTomato constructs (wild type, S354I and N352A) and labeled using anti-HA antibodies without permeabilization. Maximum projections of wide-field stacks are shown. (E) Quantification of surface HA levels relative to the tdTomato signal. Wild type, *n*=36; N354I, *n*=34; N352A, *n*=35 cells from three experiments. Each experiment is shown in a different color. Replicates are indicated by circles; triangles indicate the average of the experiment. Data are mean±s.e.m. Two-tailed *t*-test: *P*=0.00059 (wild type versus S354I), *P*=0.00099 (wild type versus N352A). Scale bars: 10 μm.

Membrane proteins require N-linked glycosylation for proper protein folding and export from the endoplasmic reticulum (ER). Fzd6 has two predicted N-linked glycosylation sites at luminal positions N38 and N352. When both sites are mutated to alanine and expressed in Cos7 or HEK293 cells, Fzd6^N38A;N352A^ is retained in the ER (Tang et al., 2020). The S354I mutation identified in *rosette* mutants alters the consensus sequence N-X-S/T, which is essential for N-linked glycosylation. The consensus sequence is found in extracellular loop two and, although it is not present in *Drosophila*, it is conserved among tetrapods suggesting functional importance (Fig. S4A). If glycosylation at N352 is inhibited in *rosette* mutants, we might expect to see a change in Fzd6 localization. Fzd6 normally localizes to the plasma membrane of basal epidermal cells, where it colocalizes with E-cadherin at intercellular junctions (Fig. 4A, top; Fig. S1A). As expected for a defect in ER export, colocalization between Fzd6 and E-Cadherin is lost in *rosette* mutants and, instead, Fzd6 localizes intracellularly to the space between the nucleus and the plasma membrane (Fig. 4A, bottom; Fig. 4B). Interestingly, although hair follicle polarity reversal occurs only in the posterior region of *rosette* embryos, we found that Fzd6 is mislocalized across the entire epidermis (Fig. 4B).

To determine whether the S354I mutation itself is responsible for Fzd6 intracellular accumulation, as opposed to another genetic element in the *rosette* background, we introduced the mutation into a Fzd6 construct with intracellular tdTomato and extracellular HA tags, and transfected control or mutant Fzd6 constructs into cultured mouse keratinocytes (Fig. 4C). By performing anti-HA staining without membrane permeabilization, we could selectively label the surface-exposed Fzd6 extracellular domain and compare the levels with total Fzd6-tdTomato. In keratinocytes expressing wild-type HA-Fzd6-tdTomato, HA labeling was abundant on the cell surface (Fig. 4D,E). By contrast, HA labeling was not detectable on the surface of cells expressing HA-Fzd6-S354I-tdTomato, and the tdTomato signal was localized to an intracellular tubular network that likely corresponds to the ER. As a control, we performed anti-HA labeling in the presence of detergent to ensure the mutant constructs functioned as expected and found that HA localized intracellularly together with tdTomato (Fig. S4B).

Next, we tested whether inhibiting glycosylation at position N352 would mimic the trafficking defect caused by the S354I mutation. We substituted asparagine 352 with alanine and performed surface HA labeling on keratinocytes expressing HA-Fzd6-N352A-tdTomato. Again, surface HA levels were not detectable and td-Tomato was localized to intracellular membranes mirroring the S354I mutation (Fig. 4D,E). These results suggest the S354I mutant likely inhibits N-linked glycosylation and that blocking the post-translational modification is sufficient to prevent the delivery of Fzd6 to the cell surface.

### The axis of PCP asymmetry rotates in the *rosette* epidermis

**Fig. 5.**
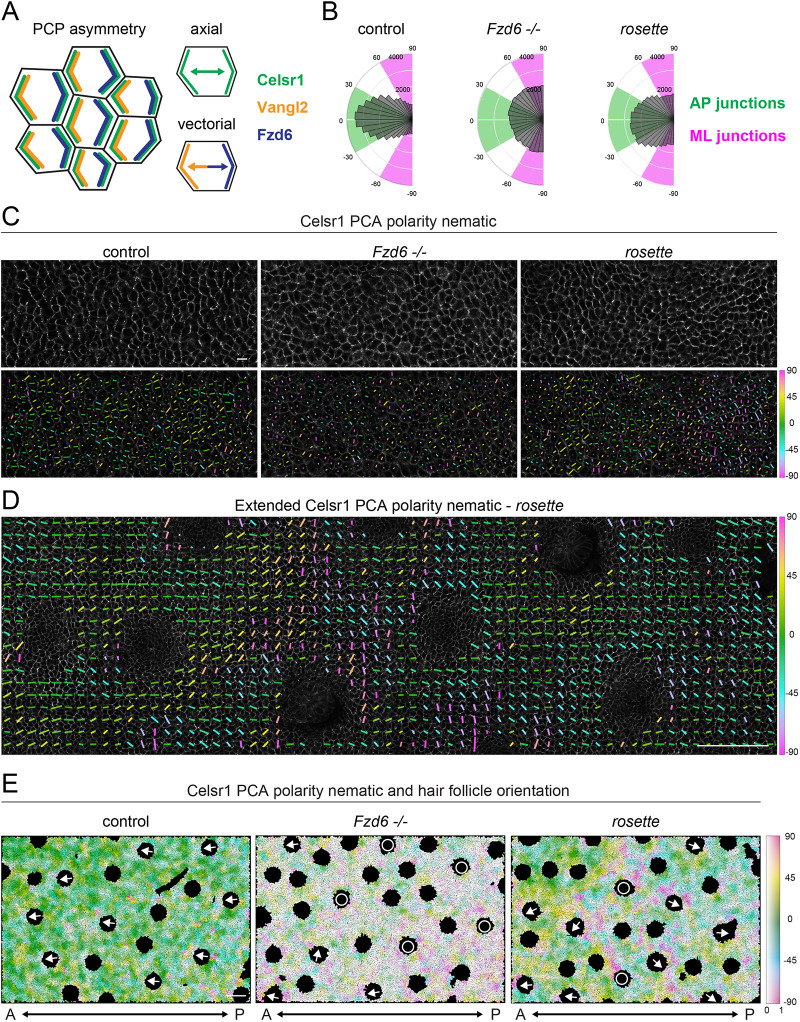
**The axis of PCP asymmetry rotates in the *rosette* epidermis.** (A) Schematic of PCP asymmetry in wild-type basal epidermal cells. (B-E) Celsr1 polarity in the basal layer of E15.5 epidermis. The areas shown are in the transition zone of *rosette* mutants and the corresponding area in *Fzd6^−/−^* and controls. (B) Circular histograms of Celsr1 polarity angles weighted by magnitude. Control, *n*=46,256 cells; *Fzd6^−/−^, n*=45,854 cells; *rosette*, *n*=48,031 cells from three embryos/genotype. (C, top) Scanning confocal images of whole-mount epidermis labeled for Celsr1. Scale bar: 10 μm. (C, bottom) Celsr1 polarity nematic calculated by PCA with QuantifyPolarity. Lines indicate the sides of the cell with the highest levels of Celsr1 and are color coded by angle (green, AP; magenta, ML). Magnitude is indicated by the length of the line, with longer lines indicating higher magnitudes. (D) Extended AP region showing the average polarity nematic for a zone of ∼10 cells. Blank spaces are newly formed follicles. Scale bar: 100 μm. (E) Spatial map of local Celsr1 polarity angle (color) and magnitude (saturation). Each cell is color-coded to represent the average angle and magnitude of a local area with a radius of 40 pixels (center cells and approximately two radii of neighbors). Black circular regions correspond to emerging hair follicles and arrows depict their orientations. White circles indicate unpolarized follicles that point straight down. Blank spaces represent newly formed follicles that are too early to categorize or areas that could not be segmented. Scale bar: 100 μm.

In the wild-type epidermis, PCP proteins are polarized along AP junctions with Celsr1 displaying axial asymmetry, forming homodimers between neighboring cells, and Vangl2 and Fzd6 displaying vectorial asymmetry, polarizing toward anterior or posterior sides of the cell, respectively (Fig. 5A) (Devenport and Fuchs, 2008). Loss of a single PCP protein leads to uniform distribution of the remaining proteins (Devenport and Fuchs, 2008; Cetera et al., 2017; Stahley et al., 2021). To understand how mislocalized Fzd6 could lead to region-specific follicle reversal in *rosette* embryos, we examined the axis of Celsr1 polarity, focusing on the transition zone, where follicles switch from anterior to posterior orientations. We used QuantifyPolarity to measure the orientation and magnitude of Celsr1 polarity based on principal component analysis (Fig. 5B, Fig. S5) (Tan et al., 2021). In wild-type embryos, Celsr1 is enriched along AP junctions (predominantly within 30° of 0°) and depleted from mediolateral (ML) junctions (within 30° of ±90°), and the magnitude of asymmetry is high. By contrast, Celsr1 is weakly polarized in no particular direction in *Fzd6^−/−^* mutants. In *rosette* mutants, Celsr1 is predominantly polarized along AP junctions but not as strongly as in wild-type controls.

To understand how Celsr1 is organized to produce this intermediate phenotype, we used QuantifyPolarity to display the polarity nematic over each cell as a line that points to the sides of the cell with the highest level of Celsr1 with the length of the line indicating the magnitude (Fig. 5C, Fig. S1A). Lines are color-coded to indicate orientation (green, AP; magenta, ML). In wild-type embryos, Celsr1 is strongly polarized along AP junctions (Fig. 5C, long green and yellow lines), whereas in *Fzd6^−/−^* embryos, Celsr1 is weakly polarized and randomly oriented (Fig. 5C, short uncoordinated lines). Interestingly, Celsr1 is strongly polarized and locally coordinated in *rosette* mutants, but the axis of polarity is not fixed. Instead, Celsr1 rotates from mainly AP junctions (Fig. 5C, long green and yellow lines) to ML junctions (Fig. 5C, long magenta lines) forming a swirling pattern, which has never been previously described in the skin. To determine whether ML enrichment resolves posteriorly, we analyzed a larger region and displayed the average polarity nematic over ∼10 cells (Fig. 5D, Fig. S1A). Celsr1 rotates from AP to ML and back to AP junctions (Fig. 5D, green, magenta, green). To determine whether the changes in the PCP axis correlate with the overall hair follicle pattern, we extended the imaging area further to encompass over 15,000 cells and 15-25 hair follicles, and generated spatial maps to represent the average axis of polarity by color and magnitude with saturation (Fig. 5E, low saturation, low magnitude; high saturation, high magnitude, Fig. S1A). In wild-type embryos, follicles are anteriorly oriented (arrows) and Celsr1 is strongly polarized along AP junctions, whereas follicles are misaligned or unpolarized (circles) in *Fzd6^−/−^* mutants and Celsr1 is weakly polarized in random orientations. In *rosette* mutants, Celsr1 asymmetry is locally aligned with high magnitudes that can be observed as highly saturated patches of cells of the same color. Although Celsr1 is polarized in many different orientations, it is often aligned along the ML axis (magenta), where follicles transition from anterior to posterior orientations. Further away from this zone, in both anterior and posterior directions, Celsr1 is predominantly polarized along AP junctions (0±40°, green-blue and green-yellow). These data suggest that in *rosette* mutants, the axis of PCP asymmetry rotates in the transition zone and is likely reversed in the posterior by a full 180°.

### PCP-directed collective cell movements are reversed in *rosette* mutants

**Fig. 6.**
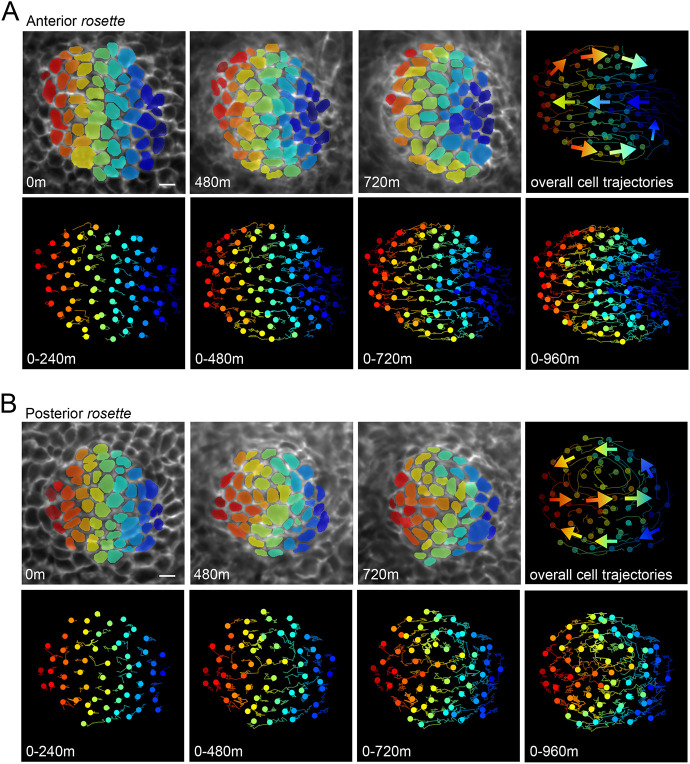
**PCP-directed collective cell movements are reversed in posterior *rosette* hair placodes.** (A,B) Spinning disk confocal images from a time series of *rosette* placode cells expressing mTomato (top). Cells were segmented and false colored in a rainbow pattern of vertical lines at t=0. Cell tracks show the movement of cells during the designated time window, with circles indicating the last position (bottom). Smoothed tracks with arrow overlays show overall movements through the course of the time series (upper right). (A) Anterior placode; see Movie 3 and additional example from another explant in Movie 4; *n*=7. (B) Posterior placode from the same explant as in A; see Movie 5 and additional example from another explant in Movie 6; *n*=9. Scale bars: 10 µm. Anterior is towards the left.

The polarized morphogenesis of the hair placode results from PCP-directed collective cell movements (Cetera et al., 2018). If the vector of PCP asymmetry in individual cells is reversed in the posterior region of *rosette* animals, such that the PCP proteins that are normally present at the anterior side of a junction are now present at the posterior side, the initial PCP-directed cell movements that establish the hair follicle tilt should also be reversed. To test this, we performed live imaging of skin explants from *rosette* mutant embryos and heterozygous controls focusing on newly formed placodes located in both anterior and posterior regions. Epithelial cells were visualized with the epidermis-specific nuclear marker K14-H2B-GFP and/or membrane-tdTomato (Muzumdar et al., 2007; Tumbar et al., 2004). Cells were tracked over ∼17 h as initially radially symmetric placodes underwent polarized cell rearrangements. In control explants, cells of the placode epithelium rearrange in a counter-rotational pattern, where posterior cells converge and migrate toward the anterior (blue cells) and anterior positioned cells move outwards and posteriorly (red and orange cells), as previously described (Fig. S6A,B, Movies 1 and 2, *n*=4 anterior, *n*=3 posterior, Fig. S1A) (Cetera et al., 2018). In the anterior region of *rosette* mutants, cell rearrangements within placodes were similar to wild-type controls (Fig. 6A, Movies 3 and 4), with the exception of one placode, where cells failed to move (*n*=7). In the posterior region, by contrast, cell rearrangements were reversed: anterior cells converged and moved towards the posterior (red, orange cells), while posterior cells were swept outwards and towards the anterior (blue cells, Fig. 6B, Movies 5 and 6, *n*=7 of 9). In two placodes located in the posterior region of *rosette* mutant explants, cell movements were directed in the correct, anterior-biased orientation, likely because they were located near the zone of reversal (*n*=2 of 9). Thus, hair follicle reversal occurs due to reversed collective cell rearrangements at the initial stages of hair follicle morphogenesis, strengthening the evidence that the *rosette* mutation causes a region-specific reversal in the vector of PCP asymmetry.

## DISCUSSION

Planar polarized structures typically orient uniformly across entire tissues. In the mammalian skin, collectively oriented hair follicles can align over distances of millimeters to meters, depending on the animal. Previous studies established that the core PCP pathway is central to the development of this organism-scale pattern, as mutations affecting the pathway in mice disrupt follicle orientation across most, if not all, of the skin surface. The *rosette* fancy mouse, a natural variant with region-specific whorls in its fur, offered a unique hair patterning defect with the potential to reveal novel mutations that alter PCP signaling and its coordination across entire tissues. We found the posterior whorls of the *rosette* fancy mouse likely develop from a 180° rotation of the PCP axis that reverses the collective cell rearrangements that orient the hair follicle, revealing that anterior and posterior regions of the skin can establish an axis of polarity independently. We determined the whorled phenotype is associated with a missense mutation in *Fzd6* that alters the consensus sequence required for N-linked glycosylation, trapping the Fzd6 protein in the ER. The Fzd6 mutation is necessary for hair follicle reversal, as overexpression of the wild-type protein restores the normal hair follicle pattern. It is unclear, however, how tissue-level mislocalization of Fzd6 can translate into a region-specific rotation of the PCP axis; further studies are required to indicate whether the Fzd6S354I mutation is sufficient to induce reversal.

### The *rosette* phenotype is distinct from other PCP mutants

The *rosette* mouse displays a unique set of PCP phenotypes that are distinct from all previously described mutants that alter epidermal planar polarity, which are comprehensively summarized in Table S4. PCP mutants generally fall into two main categories: complete or partial loss of PCP function. When PCP function is completely lost, epidermal PCP asymmetry is not established, and the directed cell rearrangements that orient embryonic hair placodes are inhibited, causing follicles to grow straight down (Basta et al., 2023; Devenport and Fuchs, 2008; Dong et al., 2018; Cetera et al., 2018). Postnatally, the initially vertical follicles rotate to form complex patterns of whorls across the body of the animal, including the rear paws (Cetera et al., 2017; Basta et al., 2023). When PCP activity is only partially compromised, owing to functional redundancy as in Fz6 knockouts, less severe epidermal phenotypes arise. Under this condition, PCP proteins also fail to polarize, but cell rearrangements in the hair placode can still occur in an uncoordinated manner to create a pattern of randomly oriented hair follicles (Cetera et al., 2018). In contrast to a complete loss of PCP, residual PCP activity causes misoriented hair follicles to rotate postnatally to correct their orientation along the AP axis, except for paw whorls, which are maintained throughout the life of the animal (Wang et al., 2010, 2006; Cetera et al., 2017).

The epidermal planar polarity defects exhibited by the *rosette* mouse cannot be fully explained as arising from a complete or partial loss of PCP function when comparing it with previously characterized PCP mutants. Notably, PCP asymmetry is established in embryonic *rosette* skin, but the axis of asymmetry is no longer coordinated across the tissue, leading to locally polarized zones that likely rotate a full 180° in the posterior of the animal. PCP-directed cell movements that orient the follicle are also reversed, resulting in a highly coordinated reversal zone. During early postnatal stages, the reversal zone is maintained, and the posterior whorls emerge. Interestingly, hairs on the rear paws of *rosette* animals are correctly oriented, suggesting PCP activity is not altered in the limbs as it is in other PCP mutants.

A key difference in epidermal polarity defects between *rosette* and PCP mutants is its region-specific reversal. Interestingly, the trafficking defect caused by the Fzd6 S354I mutation does not result in the Fzd6 null pattern of randomly oriented follicles. Furthermore, posterior-specific loss of Fzd6 activity leads to posterior-specific follicle randomization rather than reversal (Chang et al., 2016). One possibility is that a small amount of Fzd6 protein, beyond our level of detection, makes it to the membrane, resulting in a partial loss-of-function mutant that is sufficient to organize PCP along the correct axis anteriorly but insufficient to properly coordinate PCP posteriorly. This could be due to a tissue-level gradient of Fzd6 levels similar to that observed in the *Drosophila* pupal epidermis (Casal et al., 2023).

### Naturally occurring zones of polarity reversal

The opposing follicle orientations of the *rosette* mutant is reminiscent of naturally occurring reversal of planar polarized structures in other systems. Sensory hair cells of the mammalian inner ear and neuromasts of the zebrafish display ‘planar bipolarity’ in which hair cell orientation in half of the structure is reflected 180°, creating a mirror image (Kozak et al., 2020; Deans, 2021; Tarchini, 2021). In both organs, expression of the homeobox transcription factor Emx2 coincides with reversed sensory cell polarity, even though the axis of PCP asymmetry is uniform across all cells (Deans et al., 2007; Jiang et al., 2017; Holley et al., 2010; Kozak et al., 2020; Mirkovic et al., 2012; Jacobo et al., 2019). Thus, the interpretation of the PCP axis is reversed in Emx2-expressing cells.

The *Drosophila* eye disc presents another example of ‘planar bipolarity’ where ommatidia are reversed at the ‘equator’ located at the dorsal-ventral (DV) midline (Jenny, 2010; Koca et al., 2022). In contrast to the previous examples, the vector of PCP asymmetry is also reversed by 180° at the equator (Strutt et al., 2002; Rawls and Wolff, 2003). Similar to Emx2 expression in the inner ear, homeotic genes are differentially expressed in one half of the eye disc (McNeill et al., 1997; Axelrod and McNeill, 2002). However, they do not act by reversing the axis of polarity in a cell-autonomous manner, but instead define the position of the equator. Ommatidia polarity can be inverted by inducing ectopic equators or by manipulating DV patterning genes, which are thought to define the axis of PCP asymmetry as upstream directional cues (Tomlinson et al., 1997; Wehrli and Tomlinson, 1998; Zeidler et al., 1999; Cho and Choi, 1998; Domínguez and de Celis, 1998; Papayannopoulos et al., 1998; Yang et al., 2002; Rawls et al., 2002; McNeill et al., 1997; Maurel-Zaffran and Treisman, 2000; Cavodeassi et al., 1999). Taken together, planar bipolarity can be generated in two main ways: through region-specific patterns of gene expression that reverse the interpretation of the PCP axis or by reorienting the vector of PCP itself.

Our results suggest that, similar to the organization of PCP in the *Drosophila* eye, hair follicle reversal in *rosette* mutants occur because the vector of PCP in the posterior region of the skin is rotated by 180°. Although our data do not directly demonstrate a reversal of core PCP protein asymmetry, we infer that the PCP vector is flipped based on the observed rotation of junctional Celsr1 asymmetry in the transition zone where hair follicles reverse. To unambiguously resolve the vector of PCP asymmetry, super-resolution imaging of both Fzd6 and Vangl2 at epithelial cell junctions is required, which is not possible in *rosette* mutants, as Fzd6 fails to traffic to the membrane (Basta et al., 2021; Stahley et al., 2021). The observation that hair follicle orientations misalign precisely in the region where Celsr1 asymmetry is observed to rotate strongly suggests it is the axis of PCP itself, and not the interpretation of the PCP axis, that is responsible for hair follicle reversals in *rosette* mutants. However, without a readout of vectorial asymmetry, it remains possible that the PCP axis returns to its original orientation posteriorly and the interpretation of the axis is reversed.

In the mouse embryo, the Caudal-type homeobox transcription factors CDX1 and CDX2 are required for AP patterning and posterior axis elongation (van den Akker et al., 2002; Chawengsaksophak et al., 2004). They are expressed in the posterior half of the embryo and therefore are well positioned to drive posterior-specific gene expression to influence PCP in a regional manner. In fact, CDX genes regulate the expression of core PCP genes *Dvl1* and *Dvl2*, as well as the PCP regulator Ptk7 (Savory et al., 2011). Whether the reversal zone of *rosette* mutants is patterned by CDX targets will be important to determine. The proximal promoter of human CDX2 has been used to alter gene expression specifically in the posterior region of the mouse, including the skin (Hinoi et al., 2007; Chang et al., 2016; Simonson et al., 2022). The CDX2 promoter was recently used to drive posterior expression of Wnt5a, which can act as a directional cue to reorient the axis of PCP across several cell diameters (Chu and Sokol, 2016; Simonson et al., 2022). Under this condition, hair follicle orientation was altered specifically in the posterior region of the skin, but follicles were randomized rather than reversed, as we observe in *rosette* mutants (Table S4) (Simonson et al., 2022). Furthermore, Wnt5a knockout mice do not display PCP defects in the skin, and the directional cues that orient epidermal PCP remain to be discovered (Simonson et al., 2022). Whether or not posterior-specific overexpression of directional cues could reverse the axis of PCP polarity is unknown.

### Epidermal polarity reversal in natural variants

The *rosette* mutant is not the only natural variant that alters epidermal planar polarity in a distinct location. In the crested pigeon, feathers are reversed specifically around the head, whereas in the ridgeback dog, fur orientation is reversed along the posterior midline. The pigeon crest correlates with a point mutation in the kinase domain of EphB2, while the ridgeback trait is linked to a duplication of FGFs 3, 4 and 19 (Shapiro et al., 2013; Salmon Hillbertz et al., 2007). Because the mutations alter hair and feather orientation in specific regions, while the rest of the skin appears to be unaffected, we reason that planar polarity is only altered in a spatially defined zone in these breeds. What determines the precise location of the reversal zone and whether or not the mutations alter the axis of PCP asymmetry, or the interpretation of the PCP axis, remains to be investigated. The fact that different mutations alter epidermal polarity in distinct regions of the skin suggests that underlying spatial properties determine whether a zone will be refractory or sensitive to a given genetic change.

Altogether, this study provides evidence that the global axis of PCP asymmetry can be regionally decoupled across the epidermis. The missense mutation in Fzd6 present in the *rosette* mouse uncovers an ability of anterior and posterior domains to establish axes of asymmetry in opposing directions, leading to a model where a large tissue, such as the skin, uses multiple inputs at different positions to establish tissue-level patterns.

## MATERIALS AND METHODS

### Mouse lines and breeding

All procedures involving animals were approved by Princeton University and the University of Minnesota's Institutional Animal Care and Use Committee (IACUC). Mice were housed in an AAALAC-accredited facility in accordance with the Guide for the Care and Use of Laboratory Animals. Full genotypes are listed in Tables S5 and S6. Two male *rosette* animals were donated by Mike Chiodo, a fancy mouse breeder. A single male was used to rederive the line with C57BL/6 females at Charles River. Previously deceased, unrelated *rosette* animals and non-whorled fancy animals with undocumented relatedness were donated by BeeBee Mousies (Pennsylvania, USA). Animals were selected based on phenotype and availability.

### Whole-mount immunostaining

E15.5 embryos were dissected in PBS and fixed immediately in 4% paraformaldehyde in DPBS with calcium and magnesium (Gibco, 14040) for 1 h at room temperature. After washing in PBS, back skins were dissected and blocked for 1 h at room temperature or overnight at 4°C in 3% normal donkey serum, 1% bovine serum albumin, 1% fish gelatin and 0.02% sodium azide in PBT2 (PBS with 0.2% Triton X-100). Skins were incubated with primary antibodies in blocking solution overnight at 4°C then washed in PBT2. Skins were incubated with secondary antibodies for 2 h at room temperature or overnight at 4°C, washed and mounted in Prolong Gold (Molecular Probes). When the P-Cadherin antibody was used, TBS with 0.2% Triton X-100 was used instead of PBT2 for all steps. See Table S7 for antibody details.

### Quantification of embryonic hair follicle orientation

The entire back skin flanks stained for P-Cadherin and Sox9 were imaged and tiled using a Nikon A1R confocal with a Plan Apo 20×/0.75 NA objective. ‘Focus surface’ was used to find a central *z*-plane at multiple positions across the skin. Images were acquired at the central plane and 5 μm above and below. The ImageJ plugin ‘3D EDF’ was used to flatten the image (Forster et al., 2004). ImageJ and Photoshop were used for image processing.

A semi-automated method was used to detect the location and orientation of hair follicles based on the relative positions of the P-Cadherin- and Sox9-labeled cells. This produces an output image with an arrow overlay that can be hand-corrected in ImageJ to fix errors (Basta et al., 2023). The corrected output matrix is used to generate a color-coded Voronoi diagram where each polygon indicates the position of a single follicle and is colored according to its orientation.

The order parameter (Eqn 1) was calculated by taking the cosine of the difference between two follicle angles (θ_0_−θ_i_). A local area was determined by taking the average minimum distance between neighboring follicles (d) and multiplying it by 3 (3×d) such that a local area contains a center follicle and the follicles within a three-follicle radius. This step was repeated for all pairwise combinations in the field and the average was calculated. The output was scaled so the lowest value is 0 and the highest is 1 where 0 represents low order (antiparallel follicles) and 1 represents high order (parallel follicles). The center of the local area was color coded using a heat map (Kovesi, 2015 preprint) that represents the order parameter. Custom code can be found at https://github.com/rsharan2/Mouse-Epidermal-Polarity-Methods.
(1)

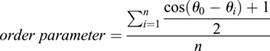


### Sample preparation and quantification of postnatal hair follicle orientation

Skins were processed as previously described (Chang et al., 2014). After euthanasia, dorsal back skin was dissected from P4 animals and pinned dermis side up to solidified paraffin wax in a petri dish. Skins were fixed in 4% PFA at 4°C overnight with gentle rocking, then washed with PBS and dehydrated over consecutive days in 70%, 95% and 100% ethanol. Dehydrated skins were placed in a glass petri dish with a glass slide laid on top to keep the tissue flat. BABB (2:1 ratio of benzyl benzoate and benzyl alcohol) was added to the dish to clear the tissue overnight with gentle rocking at room temperature. Cleared skin was placed between two glass plates for imaging. Bright-field images were acquired on a Nikon SMZ1270 dissecting scope using a Plan Apo 0.5× objective and a Nikon Digital Sight Fi1 camera. Samples were illuminated from beneath the sample using 3-6× magnification. Whole back skin images were obtained by stitching together high magnification images using Photomerge in Photoshop.

The entire dorsal skin was imaged on each animal. To account for differences in animal size from different litters, stitched images were scaled down to match the smallest skin. Thus, the same relative region of each animal was compared. Images were processed using ImageJ. To determine the orientation of each hair follicle, the plug-in ‘OrientationJ-Vector Field’ was used (Püspöki et al., 2016). The local window and grid size were adjusted (10 and 12 for a 2412×1797 pixel image) to reduce noise and allow approximately one vector to represent each follicle. This generated a vector field with the coordinates and orientation of each vector within 180°. Because our phenotypes cause complete hair follicle reversals, we needed to account for 360°. We generated a custom MATLABscript to overlay the arrows on the original image. We then generated another black and white image overlay where white indicated the vector needed to be flipped (i.e. 0 becomes 180). The updated vector field was then used to generate a color map representing the hair follicle orientation at each position, which was overlaid on the original image. The order parameter was calculated as described for the embryonic samples, except the local area analyzed was 200 pixels, including ∼200-300 follicles. Custom code can be found at https://github.com/rsharan2/Mouse-Epidermal-Polarity-Methods.

### Genome-wide SNP genotyping array

DNA was extracted from ear punches or tail clips using the Qiagen DNeasy Blood and Tissue kit. We sent high molecular weight DNA to ThermoFisher Scientific for genotyping array service using the Affymetrix Mouse Diversity Array. The array contains ∼623,000 probe sets that assay single nucleotide polymorphisms (SNPs) previously identified between mouse strains (Yang et al., 2009). We used the SNP set to identify SNPs associated with the whorled phenotype. We treated the whorled phenotype as a binary trait and categorized 91 mice (Table S1) as whorled (1) or non-whorled (0). Sample relatedness and ancestry are summarized in Table S2. After the fancy founder was outcrossed to C57BL/6 and backcrossed to phenotypic intermediates, the approximate C57BL/6 background was estimated. The original founder (E09, Table S1) and its sibling that was not used to propagate the line (H05, Table S1) are considered to have a 100% fancy background or 0% C57BL/6. Pure Black6 animals are 100% C57BL/6. We received a filtered dataset (541,069 SNP loci) after the vendor performed a quality control step during genotype cluster classification using the Axiom Analysis Suite. ‘PolyHighResolution’, ‘NoMinorHom’ and ‘MonoHIghResolution’ categories were recommended for further analysis. We applied a minor allele frequency filter (MAF) of <3% using PLINK v1.9 (Chang et al., 2015a) on unique samples that resulted in a final dataset of 67,589 SNP loci (67K SNP set). To assess the structuring of the samples with the 67K SNP set, we conducted a clustering analysis using a principle component analysis (PCA) with the program *flashPCA* (Abraham and Inouye, 2014) and PC1 captured the gradient of Black6 ancestry. The SNP array data have been deposited in GEO under accession number GSE225533.

### Genotype–phenotype association

We used a linear mixed model (*lmm*) in the program *gemma* to identify SNP variants in the 67K set associated with the binary whorl phenotype (present or absent) in the 91 samples (Zhou and Stephens, 2012). *Gemma* removes SNPs that lack information and ignores individuals that lack phenotypic data. In our sample set, 6/91 samples had an unknown phenotype and were excluded. We constructed a standardized relatedness matrix in *gemma* with the *-gk* 2 function, which was then used in the *lmm* for adjusting association scores for relatedness and used the likelihood ratio test (LRT) for significance testing. We also prioritized SNPs with allelic effect (β) values in the lower or upper 5th percentile of the distribution, and considered these SNPs as putative outliers with outlier β values. Chromosome 15 contained the highest number of SNPs that were associated with outlier β values (Table S8). The LRT identified 1428 SNPs associated with the binary whorl phenotype (*P*<10^−5^), of which all but two were located on mouse chromosome 15 (Table S9). We further identified a candidate region on chromosome 15 (25.7-62.1 Mb) containing 132 genes by integrating the LRT *P*-value and β values (Fig. 3B).

### *Fzd6* genotyping

To sequence all *Fzd6* exons, the primer sets in Table S10 were used for PCR amplification. The product was purified using the Zymo Clean and Concentrate kit and sequenced (Genewiz). To detect the *rosette* mutation, the primer sets for ‘Fzd6 exon 3 part 2’ were used. Ear punches or embryonic tails were also sent to Transnetyx for genotyping where they used the primers and reporters listed in Table S10.

### Frizzled alignment

Using NCBI protein BLAST, the mouse Fzd6 amino acid sequence was aligned to the Fzd6 sequences from rat, human, chicken, frog and Fz from *Drosophila* (Altschul et al., 1990). The multiple sequence alignment (MSA) file was downloaded and loaded into RStudio. The ggmsa package was used to display the MSA for extracellular loop 2 with each amino acid colored by its chemistry (Yu, 2022; Zhou et al., 2022).

### Colocalization of E-cadherin and Fzd6

E15.5 mouse skins stained for E-cadherin and Fzd6, and with Hoechst were imaged on an A1R scanning confocal using a Plan Apo 60×/1.4 NA objective. Single *z* planes in the interfollicular regions were chosen for analysis using only the E-cadherin and Hoechst channels. In ImageJ, a mask was created using the Auto Threshold/MinError option on the Hoechst channel. This allowed us to exclude the nuclear area in our analysis. Colocalization was measured between the E-Cadherin and Fzd6 channels, excluding the masked area, using the Coloc2 plug-in in ImageJ to determine the Pearson correlation coefficient.

### Molecular cloning and constructs

K14-HA-Fzd6-tdTomato constructs were generated by using In-Fusion (Takara) cloning to first add the tdTomato tag from pBluescript (Stratagene) to the empty K14–β-globin–MCS vector (Vaezi et al., 2002). The HA tag was inserted after the signal sequence of Fzd6 in the pCR2.1-TOPO (Invitrogen) vector by In-Fusion cloning. The S354I and N352A point mutations were introduced to HA-Fzd6 by PCR-mediated site-directed mutagenesis. The final constructs were assembled using In-Fusion cloning to insert the HA-Fzd6 fragments into the K14-tdTomato vector such that the stop sequence of Fzd6 is replaced with tdTomato. The primers used are listed in Table S11. Plasmids used have been deposited with Addgene: pCR 2.1-TOPO Fzd6HA (#208363); pCR 2.1-TOPO Fzd6 S354I HA (#208364); pCR 2.1-TOPO Fzd6 N352A HA (#208365).

### Surface detection of HA

CD1 mouse primary keratinocytes were derived from P0 dorsal skins and used for experiments within 25 passages. Cells were cultured in E-media supplemented with 15% fetal bovine serum and 0.05 mM Ca^2+^ at 37°C with 5% CO_2_ (Nowak and Fuchs, 2009). Cells were cultured on fibronectin-treated coverslips and transfected with the K14-HA-Fzd-tdTomato constructs using the Effectene transfection reagent (Qiagen). Cells were switched to high Calcium media (1.5 mM Ca^2+^) 12 h post transfection. Cells were fixed in 4% paraformaldehyde for 10 min 24 h post-transfection, washed in PBS and incubated for 1 h with the HA antibody in PBS without detergent. Cells were washed and incubated with a 647 secondary antibody for 30 min, washed and mounted in Prolong Gold.

Stained cells were imaged on a wide-field system. Positions were chosen using only the tdTomato signal with the 20× objective. A 60×/1.4NA objective was used to acquire *z* stacks at the pre-selected positions. For each cell, the exposure time was determined for the tdTomato signal and a scaling factor was determined for HA detection using the wild-type construct. For example, if a scaling factor of 2 was used for an experiment, then a 200 ms exposure time would be used for tdTomato and 400 ms for HA on cell 1 and 400 ms for tdTomato and 800 ms for cell 2. The scaling factor was kept constant for all constructs within an experiment.

Post-processing was performed in ImageJ. Max intensity projections were created for each channel and Auto-thresholding was used to make a mask on the tdTomato channel. The average intensity was calculated within the mask for each channel and the background was subtracted. The ratio of HA signal to tdTomato was calculated for each cell and the average ratio for the wild-type construct was scaled to 1 to normalize the data. Two cells transfected with the wild-type construct were omitted from the analysis because the HA signal was oversaturated.

### Spatial representation of PCP protein asymmetry

E15.5 skins stained for Celsr1 and E-cadherin were imaged on an A1R scanning confocal using a Plan Apo 60×/1.4 NA objective. *Z*-stacks were imaged and stitched together using Nikon Elements software to generate single images covering over 1 mm^2^. To merge *z*-stacks into single in-focus composite images for analysis, 3D EDF (Extended Depth of Field) (Forster et al., 2004) was used in ImageJ on individual channels. Cell masks were created using CellPose (Stringer et al., 2021) or TissueAnalyzer (Aigouy et al., 2016). Celsr1 polarity was measured using QuantifyPolarity, which outputs the cell position, magnitude and orientation of Celsr1 asymmetry (Tan et al., 2021). A custom MATLAB script was used to calculate the average vector orientation and magnitude using Eqns 22 and 23 of Tan et al. (2021) in a localized area with a radius of 40 pixels (center cells and approximately two radii of neighbors). The center cell of each neighborhood was color-coded by the average orientation with the saturation indicating the magnitude using a color map (Kovesi, 2015 preprint). The saturation histogram was scaled such that the bottom and top 1% polarity magnitude values were saturated to 0 and 1, respectively, using the ‘imadjust’ and ‘stretchlim’ functions in MATLAB. Cells with polarity magnitudes less than 0.02 were excluded. The orientation of hair follicles in the area were manually measured using the E-cadherin channel with no knowledge of Celsr1 asymmetry.

### Live imaging and cell tracking

Live imaging and cell tracking was performed as previously described (Cetera et al., 2018). E15.5 dorsal skin explants expressing membrane-tdTomato and K14-H2B-GFP were dissected in PBS and transferred onto a 1% agarose gel with F-media containing 10% fetal bovine serum, dermis side down. The agarose gel with the explants was placed into a 35 mm lummox membrane dish (Sarstedt) such that the epidermal surfaces of the explants were in contact with the membrane. Images were acquired on a Nikon Ti-E Spinning Disc with Perfect Focus using a Plan Apo 20/0.75NA air objective and 1.5× optical zoom. Explants were cultured in a humid imaging chamber at 37°C with 5% CO_2_ during imaging.

The ImageJ plug-in MultiStackReg (https://github.com/miura/MultiStackRegistration) was used to correct for XY drift, and a single *z* plane with the base of the follicle in focus was selected at each time point. Cells were manually tracked in ImageJ using the tdTomato signal and H2B-GFP, when present. Cell tracks were produced using the TrackMate plug-in (Tinevez et al., 2017). Tracks were then plotted in MATLAB, where they were false colored based on their initial AP positions. Overall cell trajectories are smoothed using the ‘smooth’ function in MATLAB with a moving average of 30 time points.

## Supplementary Material

10.1242/develop.202078_sup1 Supplementary information
