# Supplementary material for: Region-specific reversal of epidermal planar polarity in the rosette fancy mouse
Source: Development. 2023 Sep 7;150(17):dev202078. doi: 10.1242/dev.202078 (PMC10499026; doi:10.1242/dev.202078)
Supplement: Supplementary information [file develop-150-202078-s1.pdf]

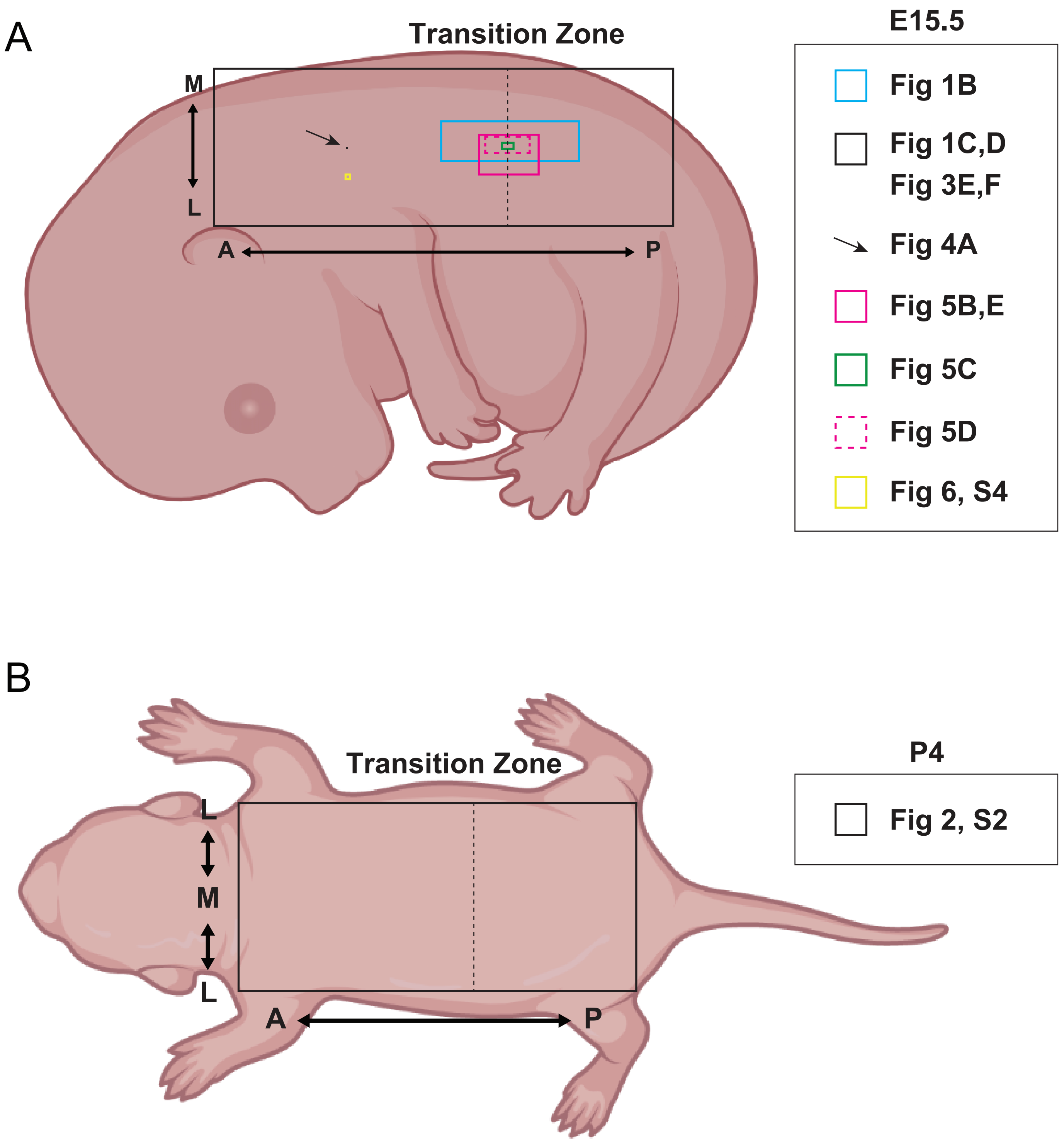

Fig. S1. Schematic of regions imaged throughout this study. (A, B) The transition zone is indicated by the black dotted line. M=medial, L=lateral, A=anterior, P=posterior. (A) Boxes indicate the relative size of imaged regions from e15.5 embryonic flanks in Figures 1B (blue), 1C, 1D, 3E, 3F (black), 4A (arrow), 5B, 5E (magenta), 5C (green), 5D (dotted magenta) 6, S4 (yellow). Multiple positions were imaged on a single flank at different locations in both anterior and posterior regions for Figures 4A, 6, S4. (B) The box indicates the area imaged for P4 pups in Figures 2 and S1. The entire dorsal skin was analyzed. Mouse images were generated by BioRender.

Development • Supplementary information

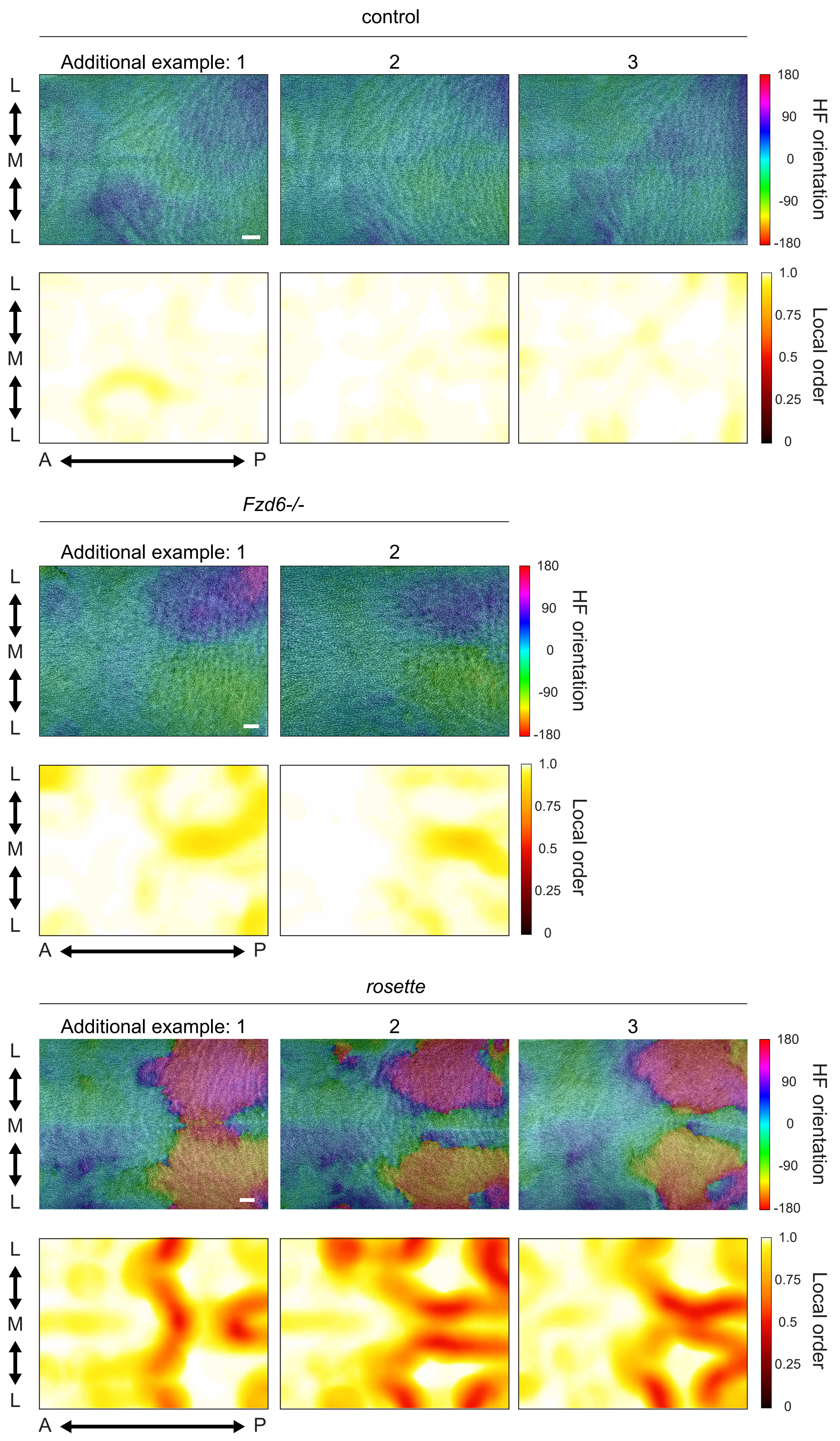

Fig. S2. Coordinated hair follicle reversal persists into postnatal stages. Additional images of cleared, flat-mounted dorsal skins at P4. Hair follicle orientation is indicated by color (cool colors= anteriorly oriented, warm colors= posteriorly oriented). Local order maps corresponding to the same images (high order= white, low order= red). AP= anterior-posterior, ML=medial-lateral with M indicating the midline. Scale bar= 1mm.

Development • Supplementary information

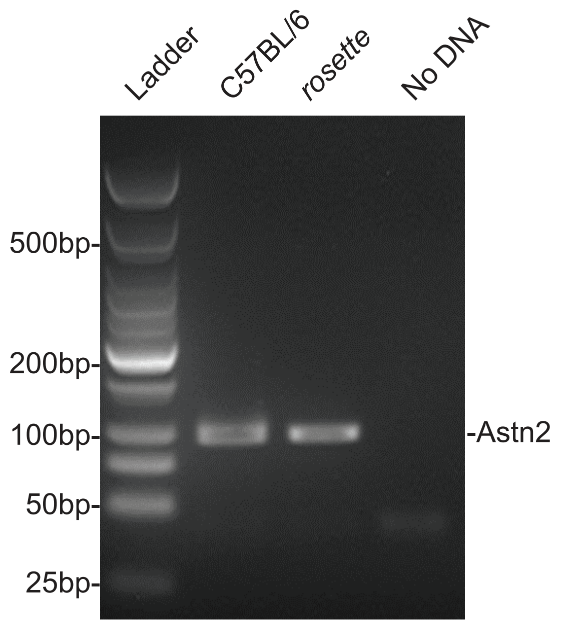

Fig. S3. The *rosette* phenotype is not due to a naturally occurring deletion in Astrotactin2. PCR product amplified from exon 5 of Astrotactin2 (Astn2). The expected 89bp product is present in C57BL/6 and *rosette* animals.

Development • Supplementary information

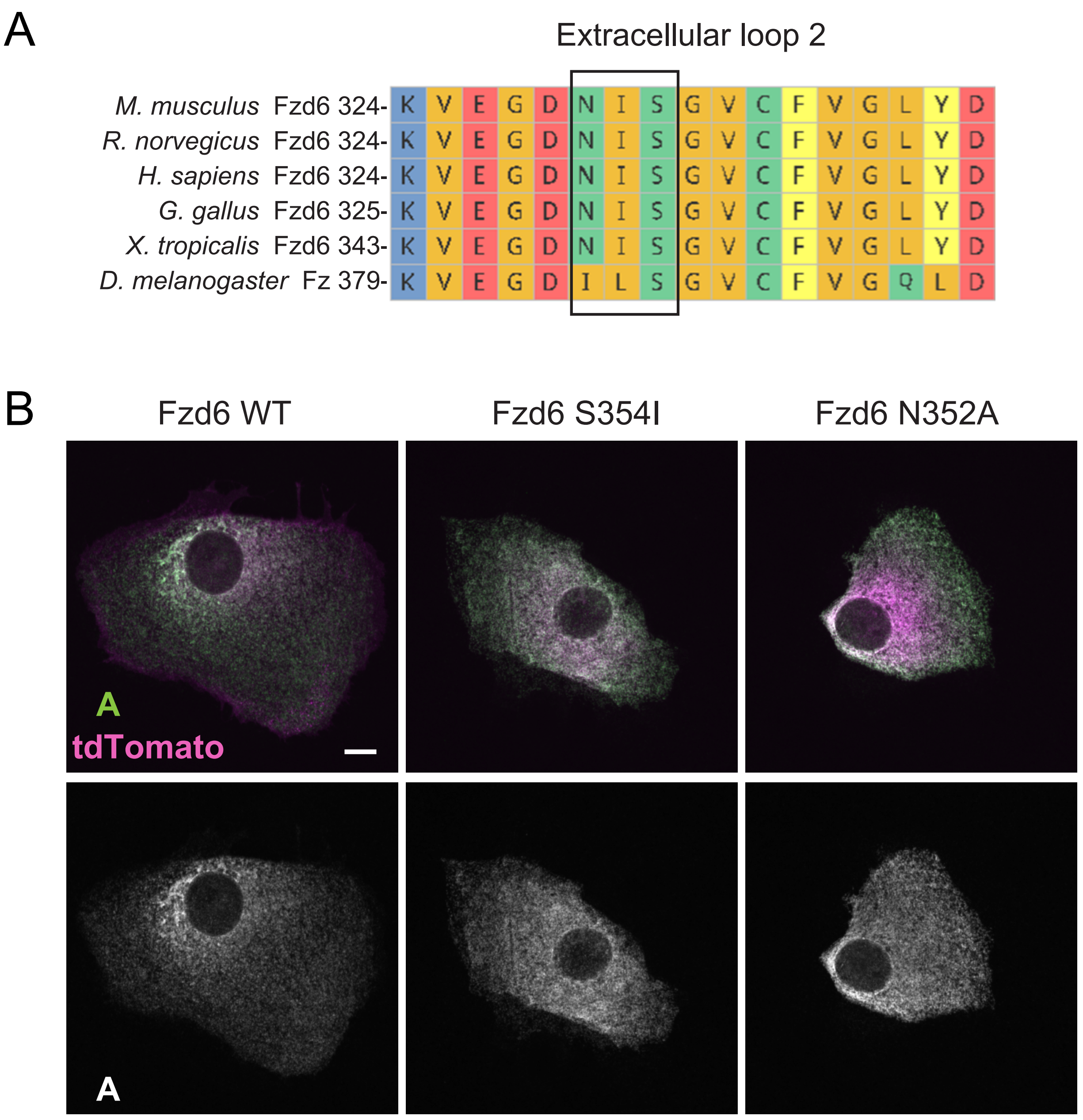

Fig. S4. The N-X-S consensus sequence is required for Fzd6 membrane localization.
(A) Alignment of Fz6 showing conservation of the N-I-S sequence (black rectangle) between mouse, rat, human, chicken, and frog. *Drosophila* Fz lacks the sequence. Amino acids are color coded by their chemistry. (B) Keratinocytes transfected with HA-Fzd6-tdTomato constructs (WT=left, S354I=center, N352A=right) and labeled with anti-HA antibodies with permeabilization. Maximum projections of wide-field stacks are shown. Scale bar, 1 microns.

Development • Supplementary information

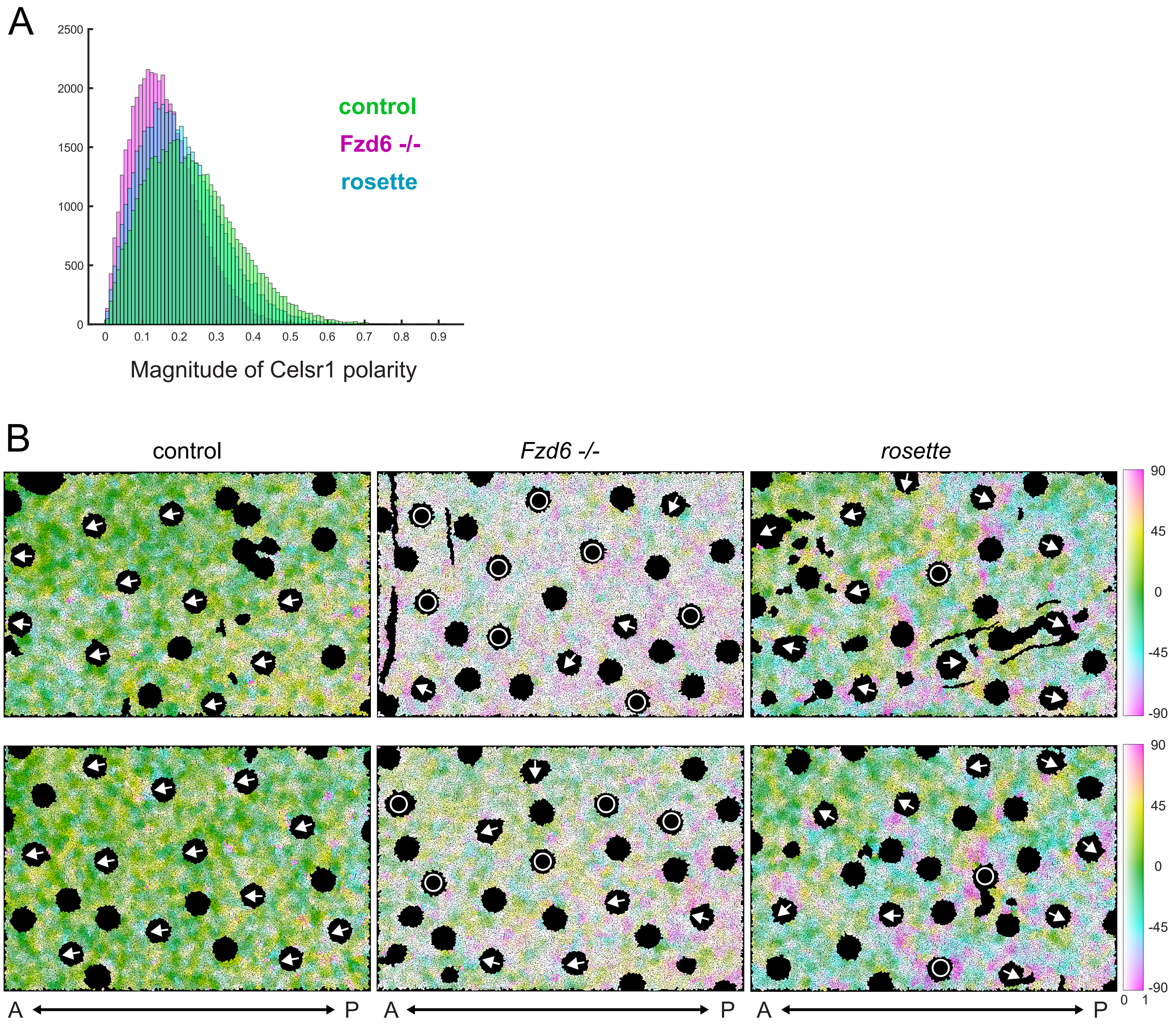

Fig. S5. The axis of PCP asymmetry rotates in the *rosette* epidermis. (A) Magnitude of Celsr1 polarity in individual cells within the transition zone of the *rosette* mutant (blue) and corresponding area in controls (green) and Fzd6-/- embryos (magenta). Control n=46,256 cells, *Fzd6-/-* n=45,854 cells, and *rosette* n=48,31 cells from 3 embryos/genotype. (B) Additional examples of long-range polarity patterns shown in Figure 5D.

Development • Supplementary information

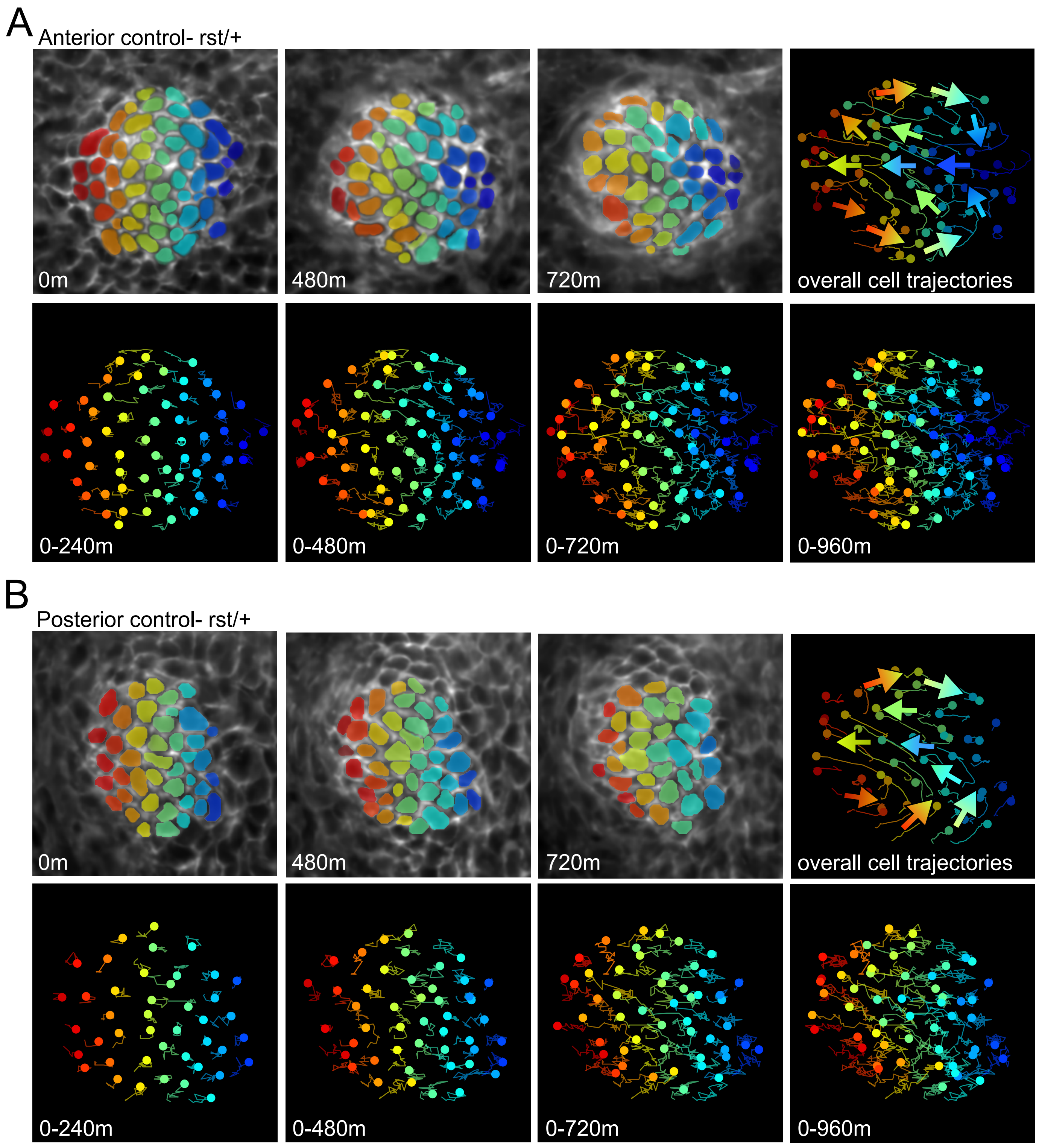

Fig. S6. PCP-directed collective cell movements occur normally in rst/+ hair placodes. Spinning disk confocal images from a time series of control *rst/+*placode cells expressing mTomato (top). Cells were segmented and false colored in a rainbow pattern of vertical lines prior to polarization. Cell tracks show the movement of cells during the designated time window with circles indicating the last position (bottom). Smoothed tracks with arrow overlays show overall movements through the course of the time series (upper right). (A) Anterior placode, see Supplemental Video. n=4. (B) Posterior placode from the same explant as (A), See Supplemental Video 2. n=3. Scale bar, 1 µm. Anterior is to the left.

Table S1. Mouse samples used for SNP analysis scored by phenotype. (Separate file) Whorled animals are represented by a “one” and non-whorled animals are represented by a “zero” in the “Phenotype” column. Familial groups are indicated along with approximate percent C57BL/6 background.

Plate ID	Mouse ID	Gender	Breed	Family-cousin (A) or sibling (A+rest)	Family-Parental	% C57BL/6	Phenotype	Duplicate ID
A01	11610-TGP1LLLRR Brn	m	fancy, C57BL/6	A		71.875	1	
A02	11610-TGP2LLLRR	m	fancy, C57BL/6	A		71.875	1	
A03	11610-TGP3LLLR	m	fancy, C57BL/6	A		71.875	1	
A04	11610-TGP4LLLL	m	fancy, C57BL/6	A		71.875	1	
A05	11610-TGP5LLRRRR	f	fancy, C57BL/6	A	I	71.875	1	
A06	11610-TGP6RRRLLL	f	fancy, C57BL/6	A	H	71.875	1	
A07	11610-TGP7LLLLRRR	f	fancy, C57BL/6	A	J	71.875	1	
A08	11610-TGP8RRRRL	f	fancy, C57BL/6	A		71.875	1	
A09	11610-TGP9RRLLL	f	fancy, C57BL/6	A		71.875	1	
A10	11610-TGP10RRRLLL	f	fancy, C57BL/6	A	G	71.875	1	
A11	11611-TGP1LLLRR brn	m	fancy, C57BL/6	A		71.875	1	
A12	11611-TGP2LLLRR	m	fancy, C57BL/6	A		71.875	1	
B01	11611-TGP3RRR	m	fancy, C57BL/6	A		71.875	1	
B02	11611-TGP4RRL	m	fancy, C57BL/6	A		71.875	1	
B03	11611-TGP5LLLRR brn	f	fancy, C57BL/6	A	F	71.875	1	
B04	11611-TGP6RRRLL	f	fancy, C57BL/6	A		71.875	1	
B05	11760-TGP1LLLRRR	m	fancy, C57BL/6	A	J	71.875	1	
B06	11760-TGP2LLLRR	m	fancy, C57BL/6	A	I	71.875	1	
B07	11760-TGP3LLLRRR	f	fancy, C57BL/6	A	E	71.875	1	
B08	11760-TGP4RRRLL	f	fancy, C57BL/6	A		71.875	1	
B09	11761-TGP1	unknown	fancy, C57BL/6	A		71.875	1	
B10	11761-TGP2	unknown	fancy, C57BL/6	A		71.875	1	
B11	11761-TGP3	unknown	fancy, C57BL/6	A		71.875	1	
B12	11761-TGP4	unknown	fancy, C57BL/6	A		71.875	1	
C01	11761-TGP5	unknown	fancy, C57BL/6	A		71.875	1	
C02	11761-TGP6	unknown	fancy, C57BL/6	A		71.875	1	
C03	11762-TGP1LLLBrn	m	fancy, C57BL/6	A		71.875	1	
C04	11762-TGP2RRL	m	fancy, C57BL/6	A		71.875	1	
C05	11762-TGP3LLLLRRRR	m	fancy, C57BL/6	A		71.875	1	
C06	11762-TGP4LLLRRR	f	fancy, C57BL/6	A		71.875	1	
C07	11610-TGN11LLLL	m	fancy, C57BL/6	A		71.875	0	
C08	11610-TGN12LLRR	m	fancy, C57BL/6	A		71.875	0	
C09	11610-TGN13RRRLL	m	fancy, C57BL/6	A		71.875	0	
C10	11610-TGN14LLLRR	m	fancy, C57BL/6	A		71.875	0	
C11	11610-TGN15LLLR	f	fancy, C57BL/6	A		71.875	0	
C12	11610-TGN16LLRR	f	fancy, C57BL/6	A		71.875	0	
D01	11610-TGN17LLLRR	f	fancy, C57BL/6	A		71.875	0	
D02	11610-TGN18RRRLL	f	fancy, C57BL/6	A		71.875	0	
D03	11611-TGN7LLLLR	m	fancy, C57BL/6	A		71.875	0	
D04	11611-TGN8RRRLL	m	fancy, C57BL/6	A		71.875	0	
D05	11611-TGN9LLLRR	m	fancy, C57BL/6	A		71.875	0	
D06	11611-TGN10LLLRR	f	fancy, C57BL/6	A		71.875	0	
D07	11611-TGN11RRRRL	f	fancy, C57BL/6	A		71.875	0	
D08	11611-TGN12LLLLR	f	fancy, C57BL/6	A		71.875	0	
D09	11611-TGN13LLLRR	f	fancy, C57BL/6	A		71.875	0	
D10	11611-TGN14RRRRL	f	fancy, C57BL/6	A		71.875	0	
D11	11760-TGN6LLLLRRR	m	fancy, C57BL/6	A		71.875	0	
D12	11760-TGN7RRRRLLL	m	fancy, C57BL/6	A		71.875	0	
E01	11760-TGN8RRL	m	fancy, C57BL/6	A		71.875	0	
E02	11760-TGN9RRRRLLL	f	fancy, C57BL/6	A		71.875	0	
E03	11760-TGN10LLLLRRR	f	fancy, C57BL/6	A		71.875	0	
E04	11760-TGN11RRRRLLL	f	fancy, C57BL/6	A		71.875	0	
E05	11762-TGN1RRR	f	fancy, C57BL/6	A		71.875	0	
E06	11762-TGN2RRLBrn	f	fancy, C57BL/6	A		71.875	0	
E07	11762-TGN3LLLRRRR	f	fancy, C57BL/6	A		71.875	0	
E08	11762-TGN4LLLLRRR	f	fancy, C57BL/6	A		71.875	0	
E09	original stud	m	fancy	B	FOUNDER	0	1	H06
E10	1031-1blkF3RRRRLLL	m	fancy, C57BL/6		A,C,D,H, K, L	62.5	1	H07
E11	105801F4RRRRLL	f	fancy, C57BL/6	C	A	81.25	0	H08
E12	105802F4LLLLRR	f	fancy, C57BL/6	C	A	81.25	0	H09
F01	10370-1RRLLLL	f	fancy, C57BL/6	D		56.25	1	
F02	10370-2LLLRRR	f	fancy, C57BL/6	D		56.25	1	
F03	10370-3LLRRRR	m	fancy, C57BL/6	D	E	56.25	1	
F04	10370-5LLLRRRR	m	fancy, C57BL/6	D	F,G	56.25	1	
F05	12070-1LLLR	m	fancy, C57BL/6	E		64.0625	1	
F06	12070-2LLLLRRR	f	fancy, C57BL/6	E		64.0625	1	
F07	12070-3LLLLRRRR	f	fancy, C57BL/6	E		64.0625	1	
F08	12070-4RRR	f	fancy, C57BL/6	E		64.0625	1	
F09	12070-5RRRRLLL	f	fancy, C57BL/6	E		64.0625	1	
F10	12070-6LLRRBrn	f	fancy, C57BL/6	E		64.0625	1	
F11	12090-1LLRRR	m	fancy, C57BL/6	F		64.0625	1	
F12	12100-1RRL	m	fancy, C57BL/6	G		64.0625	1	
G01	12100-2RRRRLLL	m	fancy, C57BL/6	G		64.0625	1	
G02	12100-3RRLLLL	f	fancy, C57BL/6	G		64.0625	1	
G03	12100-4RRRLL	f	fancy, C57BL/6	G		64.0625	1	
G04	12100-5LLLRR	f	fancy, C57BL/6	G		64.0625	1	
G05	12120-1RRRRLLLL	m	fancy, C57BL/6	H		67.1875	1	
G06	12120-2RRR	f	fancy, C57BL/6	H		67.1875	1	
G07	12120-3RRLBrn	f	fancy, C57BL/6	H		67.1875	1	
G08	12120-4LLLLRRR	f	fancy, C57BL/6	H		67.1875	1	
G09	12120-5LLLR	f	fancy, C57BL/6	H		67.1875	1	
G10	12130-1RRLLL	m	fancy, C57BL/6	I		71.875	1	
G11	12130-2RRRLL	m	fancy, C57BL/6	I		71.875	1	
G12	12150-1LLLLRRR	f	fancy, C57BL/6	J		71.875	1	
H01	12151-1RRRLL	m	fancy, C57BL/6	J		71.875	1	
H02	10390-1LLLLR	f	fancy, C57BL/6	K		56.25	1	
H03	10720-1LRRRR	m	fancy, C57BL/6	L		56.25	1	
H04	10720-2LLLLR	m	fancy, C57BL/6	L		56.25	1	
H05	original brother	m	fancy	B		0	1	
H06	original stud	m	fancy	B	FOUNDER	0	1	E09
H07	1031-1blkF3RRRRLLL	m	fancy, C57BL/6		A,C,D,H, K, L	62.5	1	E10
H08	105801F4RRRRLL	f	fancy, C57BL/6	C	A	81.25	0	E11
H09	105802F4LLLLRR	f	fancy, C57BL/6	C	A	81.25	0	E12
H10	B6 female	f	C57BL/6			100	0	H11
H11	B6 female	f	C57BL/6			100	0	H10
H12	B6 male	m	C57BL/6			100	0	

Development • Supplementary information

Table S2. Summary of mouse sample familial groups and approximate C57BL/6 ancestry.

The number of samples from each familial group is indicated under "N."

Sibship	Percent. C57BL/6 ancestry	N
B (Fancy founder)	0	1
B (Sibling of fancy founder; not crossed)	0	1
A	71.9	56
C	81.3	2
D	56.3	4
E	64.1	6
F	64.1	1
G	64.1	5
H	67.2	5
I	71.9	2
J	71.9	2
K	56.3	1
L	56.3	2
Parental animal to A, C, D, H, K, L	62.5	1
C57BL/6 (Lab strain)	100	2

Table S3. *Fzd6* SNP variants between C57BL/6 and rosette determined by Sanger sequencing. Two mutations are silent (green), one is a known, missense SNP variant (blue), and one is a novel missense mutation (magenta).

Type of mutation	Mouse strain	DNA sequence	Amino acid sequence
Missense mutation, novel SNP	C57BL/6	1048-GGAGACAACATTA**G**CGGC	350-GDNI**S**G
	rosette	1048-GGAGACAACATTA**T**CGGC	350-GDNI**I**G
Silent mutation, SNP variant	C57BL/6	1078-GGCCT**G**TATGACCTGGAC	360-G**L**YDLD
	rosette	1078-GGCCT**A**TATGACCTGGAC	360-G**L**YDLD
Silent mutation, SNP variant	C57BL/6	1858-GGGCCCAG**T**TCCAAGCTC	620-GP**S**SKL
	rosette	1858-GGGCCCAG**C**TCCAAGCTC	620-GP**S**SKL
Missense mutation, SNP variant	C57BL/6	1873-CTCTCTGGG**A**ACCGGAAC	625-LSG**N**RN
	rosette	1873-CTCTCTGGG**G**ACCGGAAC	625-LSG**D**RN

Development • Supplementary information

Table S4. Summary of PCP and *rosette* mutant phenotypes. Comprehensive summary of differences between previously characterized PCP mutants and *rosette*.

Mutant	Lethality	PCP polarization	Hair follicle pattern: Embryonic	Hair follicle pattern: Early postnatal (P2-3)	Hair follicle pattern: Late postnatal (>P6)	Hair follicle pattern: Region specific	Hair follicle pattern: Paw whorl	Reference
rosette	No	Yes	Posterior reversal	Posterior reversal	Posterior whorls	Yes	No	This paper
Fzd6-/-	No	No	Randomized/ straight down	Randomized	Recovers	No	Yes	Guo et al, 2004; Wang et al, 2006; Wang et al, 2010 ; Cetera et al, 2017
Fzd3-/-	Early postnatal lethal	–	No phenotype	NA	NA	No	–	Wang et al, 2002; Dong et al 2018
Fzd6-/-; Fzd3 -/-	Open neural tube	–	Straight down	NA	NA	No	NA	Dong et al 2018
Fzd6-/-; Astn2 ex5del/del	No	–	–	Randomized	Whole body whorls	No	–	Chang et al, 2015
Vangl2 lp/lp	Open neural tube	No	Straight down	NA	NA	No	NA	Devenport and Fuchs, 2008
Celsr crsh/crsh	Open neural tube (background dependent)	No	Straight down	–	Whole body whorls	No	Yes	Devenport and Fuchs, 2008; Ravni et al, 2009
Celsr1-/-	Open neural tube (occasional)	No	Straight down	–	Variable whorls	No	Yes	Basta et al, 2023
Wnt5a-/-	Early postnatal lethal	–	No phenotype	NA	NA	No	–	Yamaguchi et al, 1999; Simonson et al, 2022
**Skin-specific knockouts**								
Fzd3; Fzd6 dcKO	No	–	–	–	Recovers	No	–	Dong et al 2018
Vangl2 cKO	No	No	Randomized/ straight down	Randomized	Recovers	No	Yes	Cetera et al, 2017
Vangl1; Vangl2 dcKO	No	No	Straight down	–	Whole body whorls	No	Yes	Chang et al, 2016; Cetera et al, 2017
**Region-specific knockouts**								
Posterior *Fzd6cKO*	No	–	Posterior randomization	Posterior randomization	–	Yes	–	Chang et al, 2016
Anterior *Fzd6cKO*	No	–	Anterior randomization	Anterior randomization	–	Yes	–	Chang et al, 2016
Posterior *Vangl1; Vangl2 dcKO*	Spina bifida; postnatal lethal	–	–	Posterior randomization	NA	Yes	–	Chang et al, 2016
Posterior *Wnt5a cKO*	No	–	No phenotype	No phenotype	–	No	–	Simonson et al, 2022
**Region-specific over expression**								
Posterior *Wnt5a OE*	No	No*	Posterior randomization/ straight down	Posterior randomization	–	Yes	–	Simonson et al, 2022

– = Not reported

* = No asymmetry detected in mutant or controls

Table S5. Mouse genotypes.

Figures	Stages	Genotypes
**1**	Adult, E15.5	*C57BL/6,*<br>*Fzd6 -/-,*<br>*rosette/rosette*
**2**	P4	*rosette/+,*<br>*Fzd6 -/-*,*<br>*rosette/rosette*
**3A-C**	Postnatal	**See Table S1**
**3D**	Postnatal	*Unrelated whorled and non-whorled fancy mice*
**3E**	E15.5	*Fzd6 +/-,*<br>*rosette/+,*<br>*Fzd6 +/-; rosette/+*
**3F**	E15.5	*Rosa26 lox-stop-lox Fzd6WT; rosette/rosette,*<br>*K14-Cre; Rosa26 lox-stop-lox Fzd6WT; rosette/rosette*
**4A**	E15.5	*C57BL/6,*<br>*rosette/rosette*
**5**	E15.5	*C57BL/6,*<br>*Fzd6 -/-,*<br>*rosette/rosette*
**6**	E15.5	*K14-Cre; ROSA26 mTmG; K14-H2BGFP/+; Fzd6 rosette/+,*<br>*K14-Cre; ROSA26 mTmG; Fzd6 rosette/+,*<br>*K14-Cre; ROSA26 mTmG; K14-H2BGFP/+; Fzd6 rosette/rosette,*<br>*K14-Cre; ROSA26 mTmG; Fzd6 rosette/rosette*

*P4 *Fzd6 -/-* whole skin images were reused from Cetera, 2017.

Table S6. Mouse allele source.

Allele	Source
C57BL/6J	JAX 000664
Fzd6 -/-	Guo et al. 2004 (JAX 012824)
rosette/rosette	Breeding community
Rosa26 lox-stop-lox Fzd6WT	Hua et al. 2014 (JAX 023843)
K14-Cre	Vasioukhin et al. 1999
ROSA26 mTmG	Muzumdar et al. 2007 (JAX 007676)
K14-H2BGFP	Tumbar et al. 2004

Development • Supplementary information

Table S7. Antibodies.

Antibody	Source	Concentration
Guinea pig anti-Celsr1, polyclonal	D. Devenport (Devenport and Fuchs, 2008)	1:1000
Rat anti-E-cadherin, monoclonal	ThermoFisher, DECMA-1 clone, 14-3249-82	1:1000
Rat anti-P-Cadherin, monoclonal	Clontech, PCD-1 clone, M109	1:200
Rabbit anti-Sox9, polyclonal	Millipore, AB5535	1:1000
Goat anti-Fzd6, polyclonal	R&D Systems, AF1526	1:400
Rat anti-HA, monoclonal	Roche, 3F10 clone, 1867423001	1:250-1:500
Hoechst	Invitrogen, H1399	1:1000
Donkey anti-guinea pig 647	Invitrogen, A211450	1:1000
Donkey anti-rat 488	Invitrogen, A21208	1:1000
Donkey anti-goat 555	Invitrogen, A21432	1:1000
Donkey anti-rabbit 555	Invitrogen, A31572	1:1000
Donkey anti-rat 647	Jackson ImmunoResearch, 712605153	1:1000-1:2000

Table S8. Summary of linear mixed model for genetic associations with the binary whorl phenotype. Bolded values indicate a chromosomal enrichment of outlier ß values.

Chr	N_{SNPs}	SNP density (Kb)	N_{SNPs} with outlier ß values	Prop. of chromosome with outlier ß values
1	9,698	19.8	683	10
2	1,259	38.5	628	10
3	1,721	88.8	95	1
4	5,281	28.4	902	14
5	6,215	23.8	786	12
6	2,631	55.3	49	1
7	4,788	29.7	18	0
8	5,344	23.5	64	1
9	4,860	24.5	31	0
10	2,571	48.8	6	0
11	1,340	86	24	0
12	2,346	46.7	108	2
13	2,826	40.5	819	13
14	371	261.6	18	0
**15**	**4,976**	**20.2**	**2,092**	**32**
16	2,938	30.8	230	4
17	44	1,992	12	0
18	3,792	23.1	58	1
19	662	86.6	172	3
20	1,016	157.8	0	0

Development • Supplementary information

Table S9. Linear mixed model association for 91 mice (85 with known binary whorl phenotypes) with 67K SNPs. (Separate file) Annotations for each SNP is provided, which includes the physical gene name associated, annotated feature, and predicted phenotypic impact. (Abbreviations: AF, allele frequency; L_remle, restricted maximum likelihood estimate of lambda; L_mle, maximum likelihood estimate of lambda; N_miss, number of missing loci; p_LRT, p-value from the likelihood ratio test; S.E., standard errors for ß values).

Click here to download Table S9

Table S10. Genotyping primers.

Name	Sequence
Fzd6 exon1 For	CGGGCAGGTGGATATGTCTCTTGCT
Fzd6 exon1 Rev	ACAGCGGGAGGAGGGGAACTTAATG
Fzd6 exon2 For	TGGGATGCATGAACAATGACAAAGC
Fzd6 exon2 Rev	ACCGTGCCTACCTATGAGGGTGGAG
Fzd6 exon3 For	GAGAGGAGTGAAGGGAAGGGGGAAT
Fzd6 exon3 Rev	CCCAGCAAAAACCCCACGAAGTACA
Fzd6 exon3 part2 For	ACGATTCAGATACCCAGAGAGACCAA
Fzd6 exon3 part2 Rev	GAAGAAAGCACTCCTGGGCCAGTCC
Fzd6 exon4 For	GCCGAGCCATTTTTGTAGCCCCTAA
Fzd6 exon4 Rev	TGGTGCTAAAAGTAAGTTCTGCTTGTCCA
Fzd6 exon5 For	TGGACTTAGGGCAAAGGTTGTGTCTG
Fzd6 exon5 Rev	GCCAGGAAAAGATGAGTAACGCTCTCC
Fzd6 exon6 For	TGTGGTTTGAAGGGCTACCCAGGAG
Fzd6 exon6 Rev	TTCCTTTTGCAGTGCTGGGAGACAA
Fzd6 Transnetyx For	GCTCGCTATGAATAAGGTTGAAGGA
Fzd6 Transnetyx Rev	CGAGAGGCGTCCAGGTCATA
Fzd6 Transnetyx Reporter 1	CAAACGCCGCTAATGT
Fzd6 Transnetyx Reporter 2	AAACGCCGATAATGT
Astn2 exon5 For	AATCAGCTTTGGGAGAACCA
Astn2 exon5 Rev	GACTGCGGTACTGCTCTGTG

Table S11. Primers for generating Fzd6 constructs.

Primer	Sequence
Infusion K14 For	GTCGAGAGATCTCCGCGGTATCTAGAGTCC
Infusion K14 Rev	TGGCGGCGGATCCGTATTCG
Infusion tdTom For	ACGGATCCGCCGCCAGAGCTCCACCGCGGTGGC
Infusion tdTom Rev	CGGAGATCTCTCGACTTACTTGTACAGCTCGTCCATGCCGTACAG G
HA insertion For	CGTAAGAGGACACAGCCTTTTCTACCCATACGATGTTCCAGATTAC GCTACCTGTGAGCCAATCACC
HA insertion Rev	AGCGTAATCTGGAACATCGTATGGGTAGAAAAGGCTGTGTCCTCTT ACGAGG
Fzd6 S354I For	AATAAGGTTGAAGGAGACAACATTATCGGCGTTTGCTTC
Fzd6 S354I Rev	GAAGCAAACGCCGATAATGTTGTCTCCTTCAACCTTATT
Fzd6 N352A For	AATAAGGTTGAAGGAGACGCCATTAGCGGCGTTTGCTTC
Fzd6 N352A Rev	GAAGCAAACGCCGCTAATGGCGTCTCCTTCAACCTTATT
Infusion K14tdTom For	ATGGTGAGCAAGGGCGAGGAG
Infusion K14tdTom Rev	TGGCGGCGGATCCGT
Infusion HA Fzd6 For	ACGGATCCGCCGCCAATGGAAAGGTCCCCGTTTCTGT
Infusion HA Fzd6 Rev	GCCCTTGCTCACCATAGCGTCGGAATGGCTGCCAGCAC

Development • Supplementary information

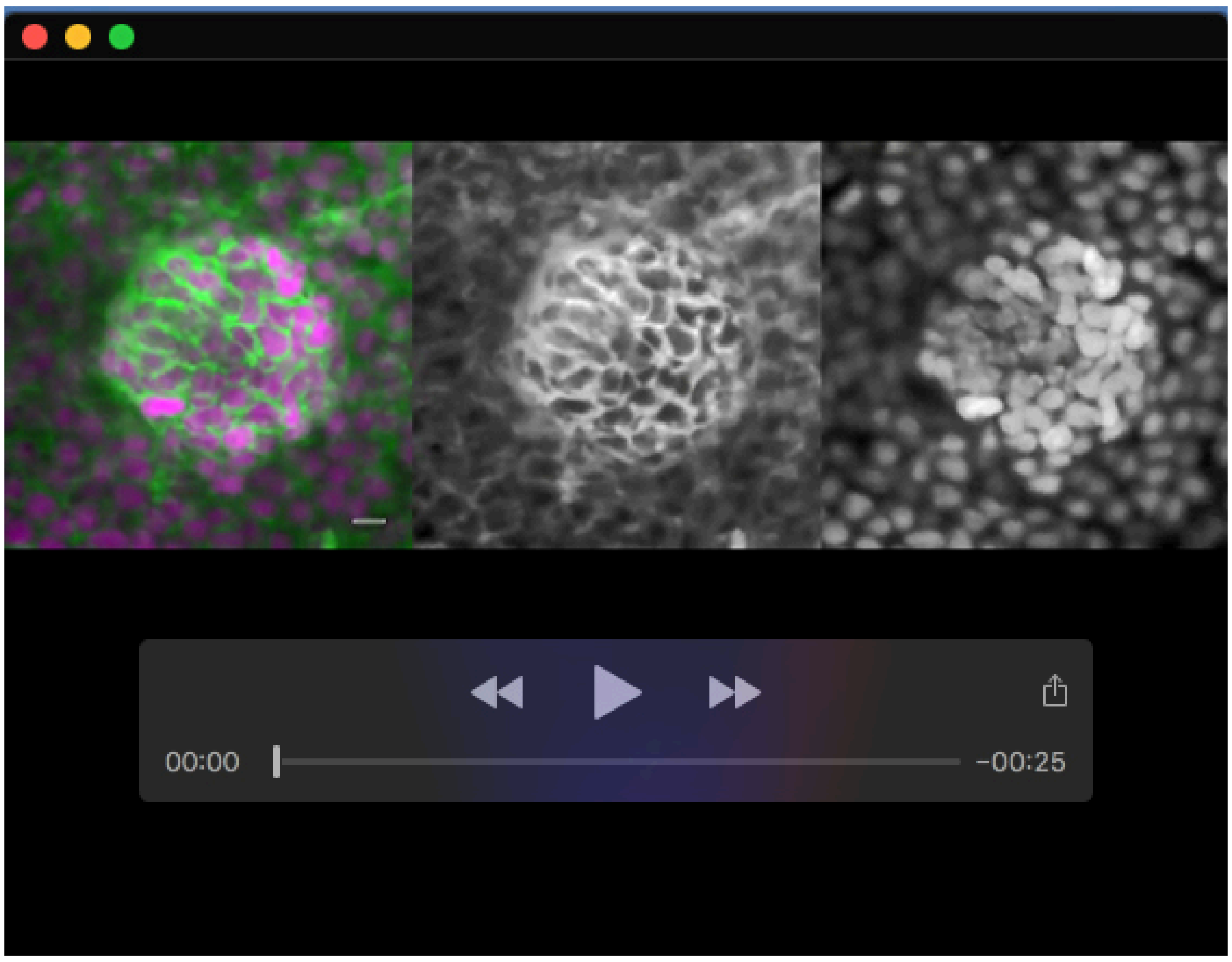

Movie 1. PCP-directed collective cell movements occur normally in control explants. Live imaging of the anterior control placode tracked cells in Figure S4A. Epidermal nuclei are shown in magenta (H2BGFP) and cell membranes are shown in green (mTomato). 17 hours/ 20 minute interval. Scale bar, 10 µm. Anterior is to the left.

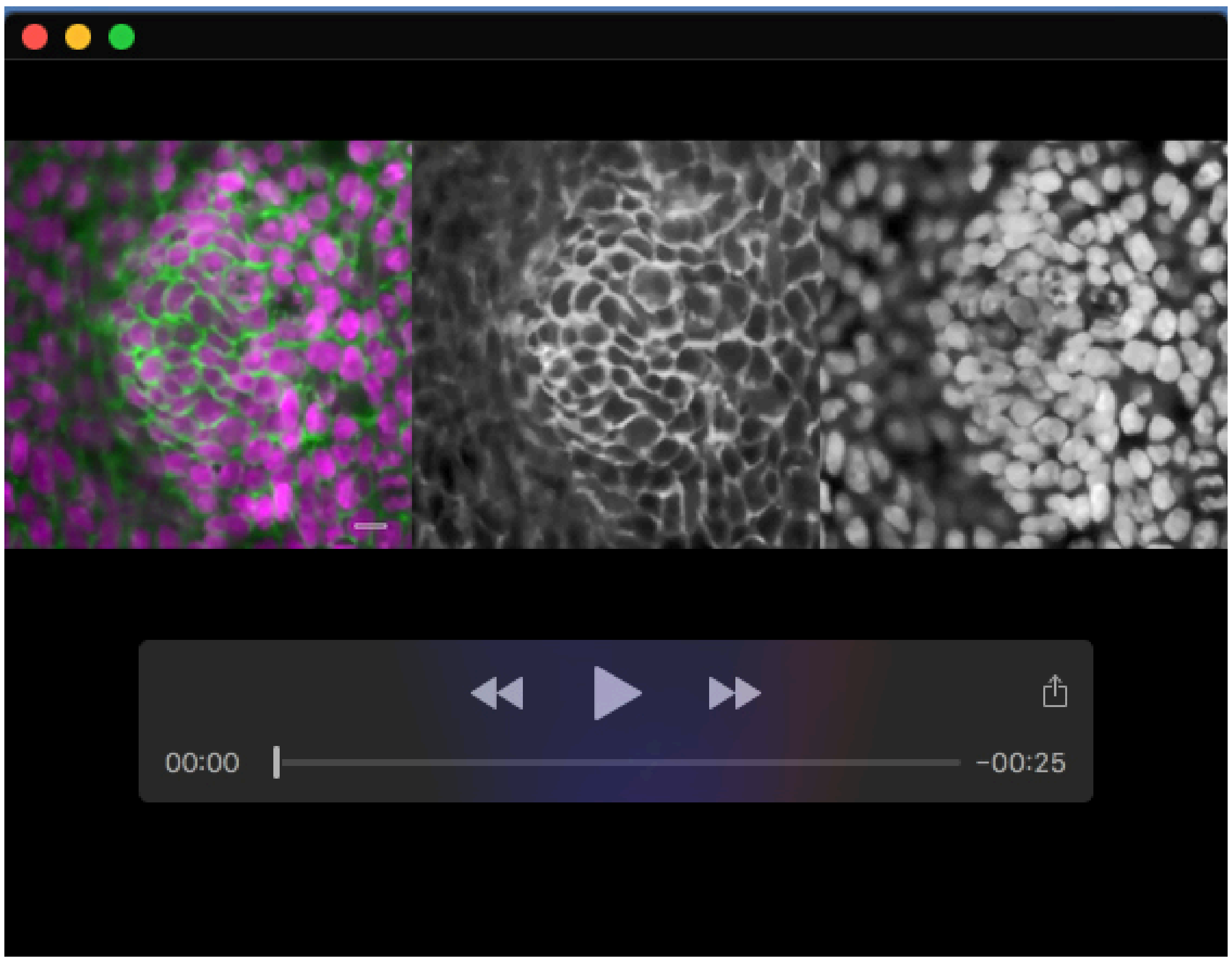

Movie 2. PCP-directed collective cell movements occur normally in control explants. Live imaging of the posterior control placode tracked cells in Figure S4B. Nuclei are shown in magenta (H2BGFP) and cell membranes are shown in green (mTomato). 17 hours/ 20 minute interval. Scale bar, 10 µm. Anterior is to the left.

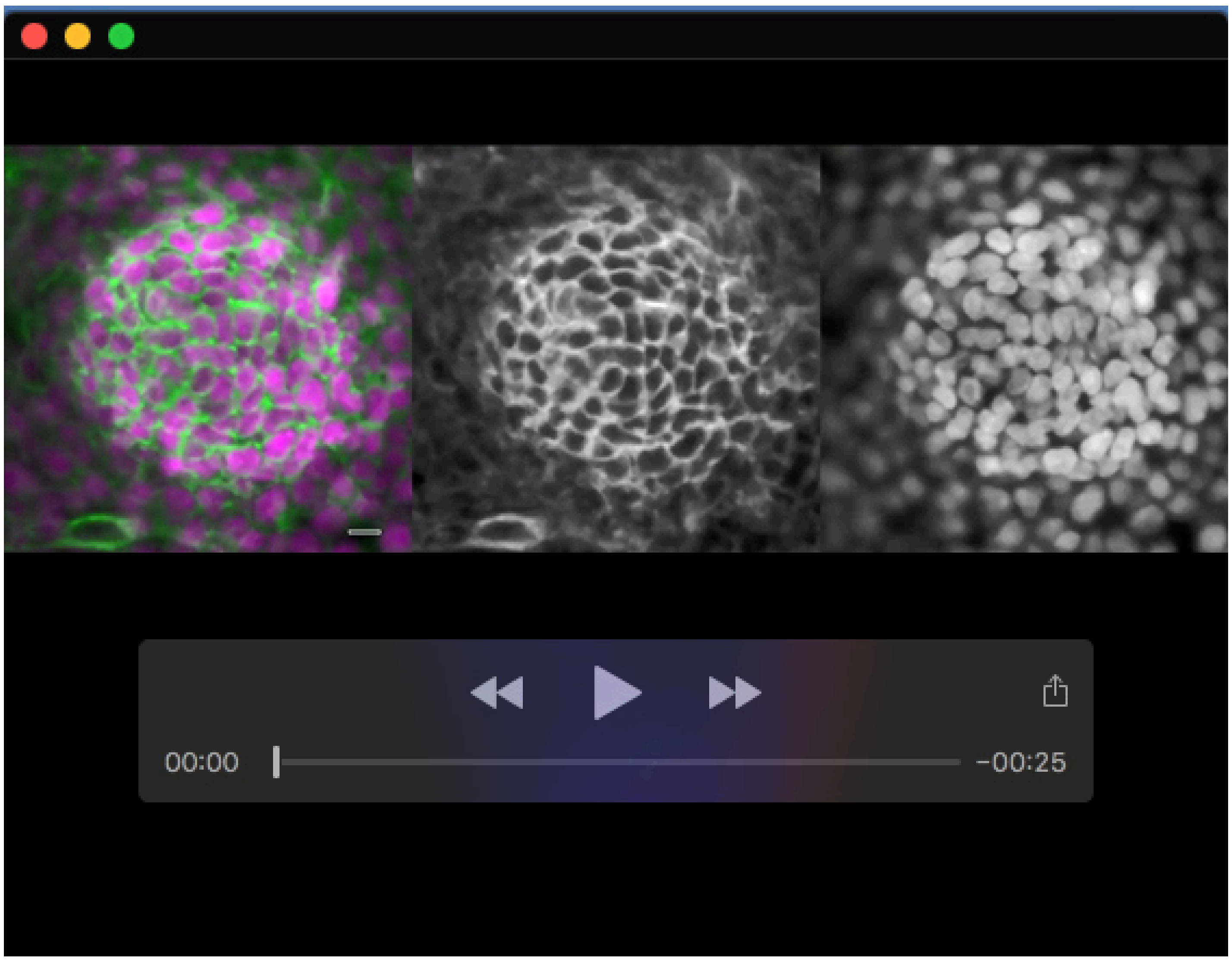

Movie 3. PCP-directed collective cell movements occur normally in anterior *rosette* placodes. Live imaging of the anterior *rosette* placode tracked cells in Figure 6A. Nuclei are shown in magenta (H2BGFP) and cell membranes are shown in green (mTomato). 17 hours/ 20 minute interval. Scale bar, 10 µm. Anterior is to the left.

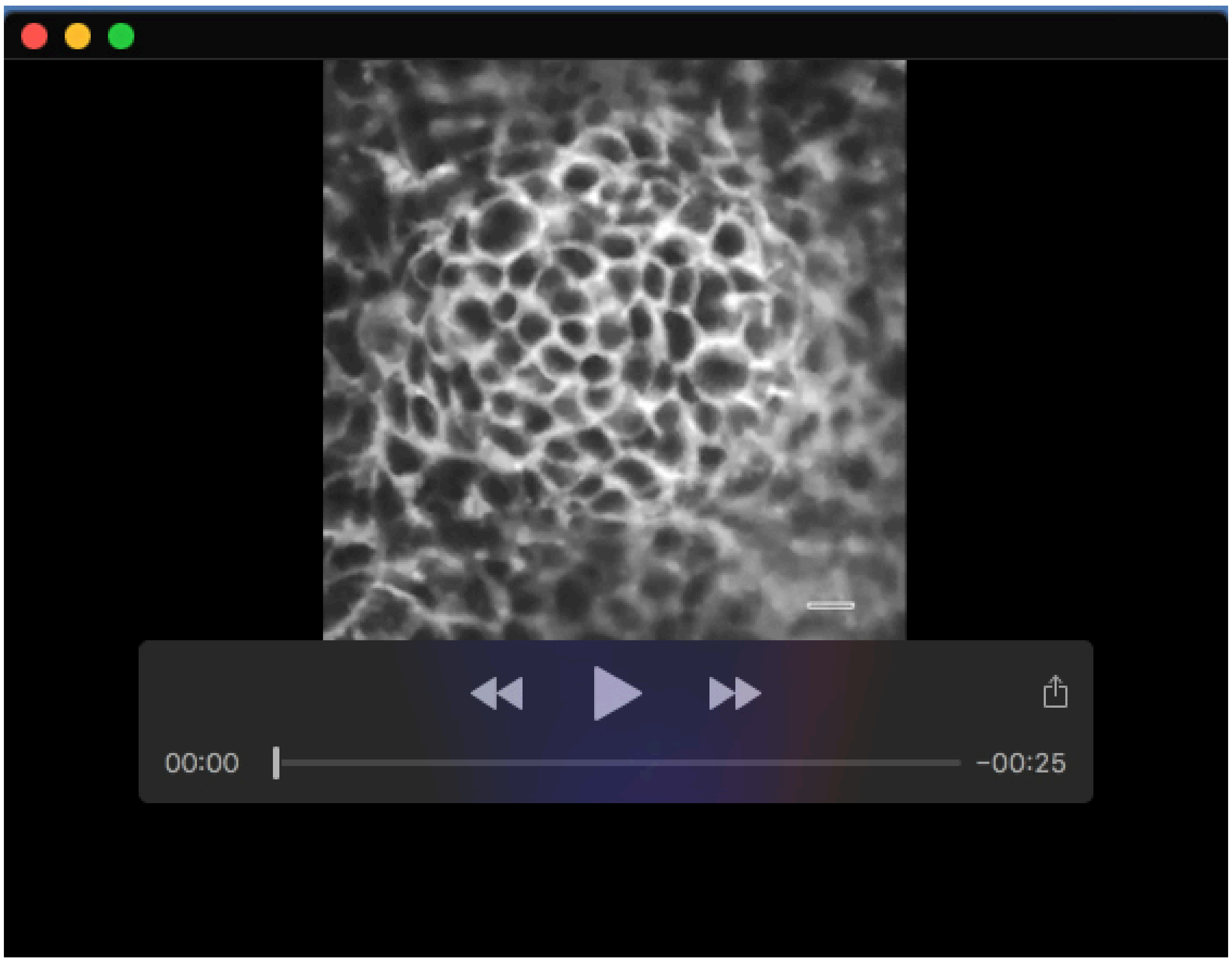

Movie 4. PCP-directed collective cell movements occur normally in anterior *rosette* placodes. Live imaging of an additional anterior *rosette* placode expressing mTomato to label cell membranes. 17 hours/ 20 minute interval. Scale bar, 10 µm. Anterior is to the left.

Development • Supplementary information

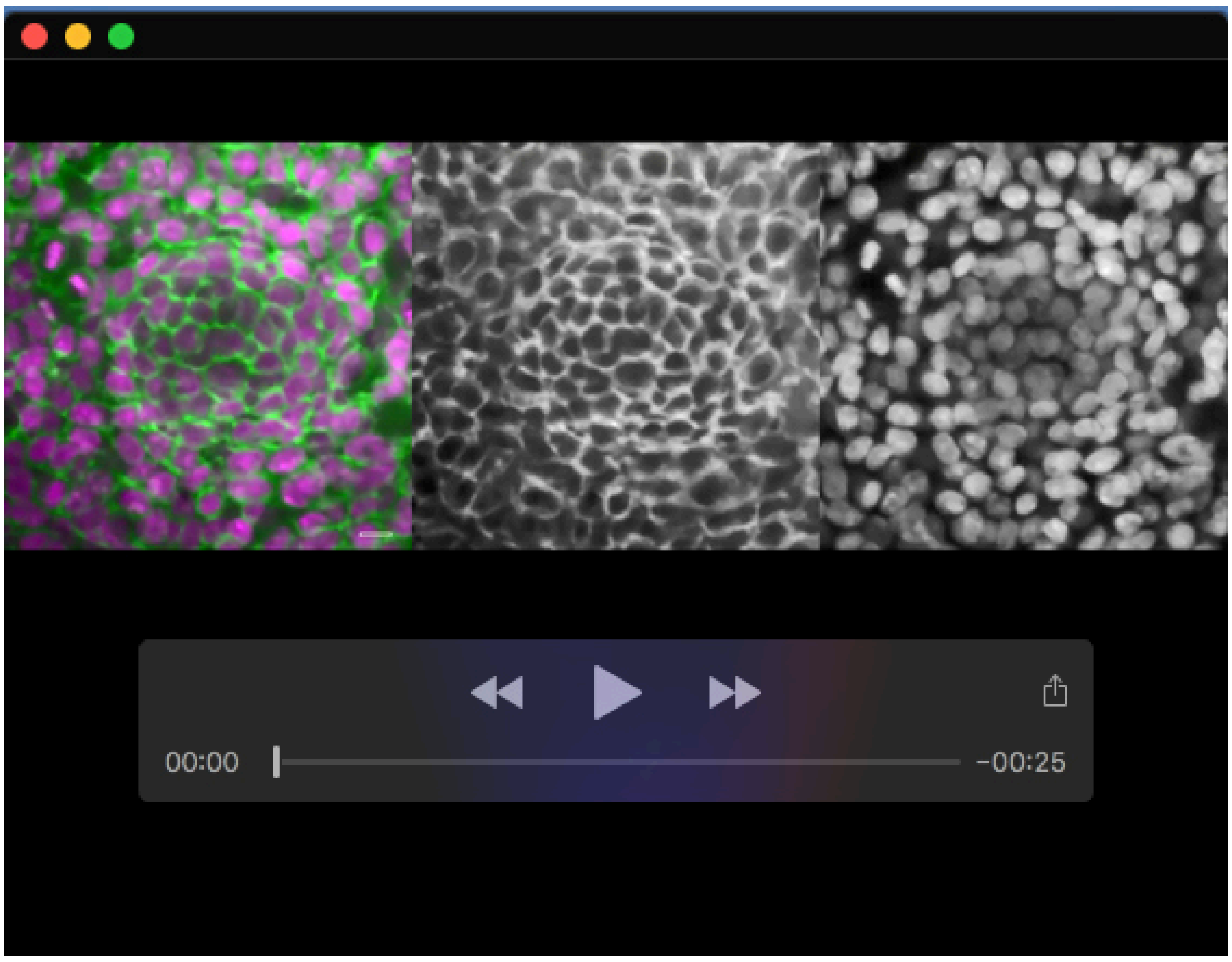

Movie 5. PCP-directed collective cell movements are reversed in posterior *rosette* placodes. Live imaging of the posterior *rosette* placode tracked cells in Figure 6B. Nuclei are shown in magenta (H2BGFP) and cell membranes are shown in green (mTomato). 17 hours/ 20 minute interval. Scale bar, 10 µm. Anterior is to the left.

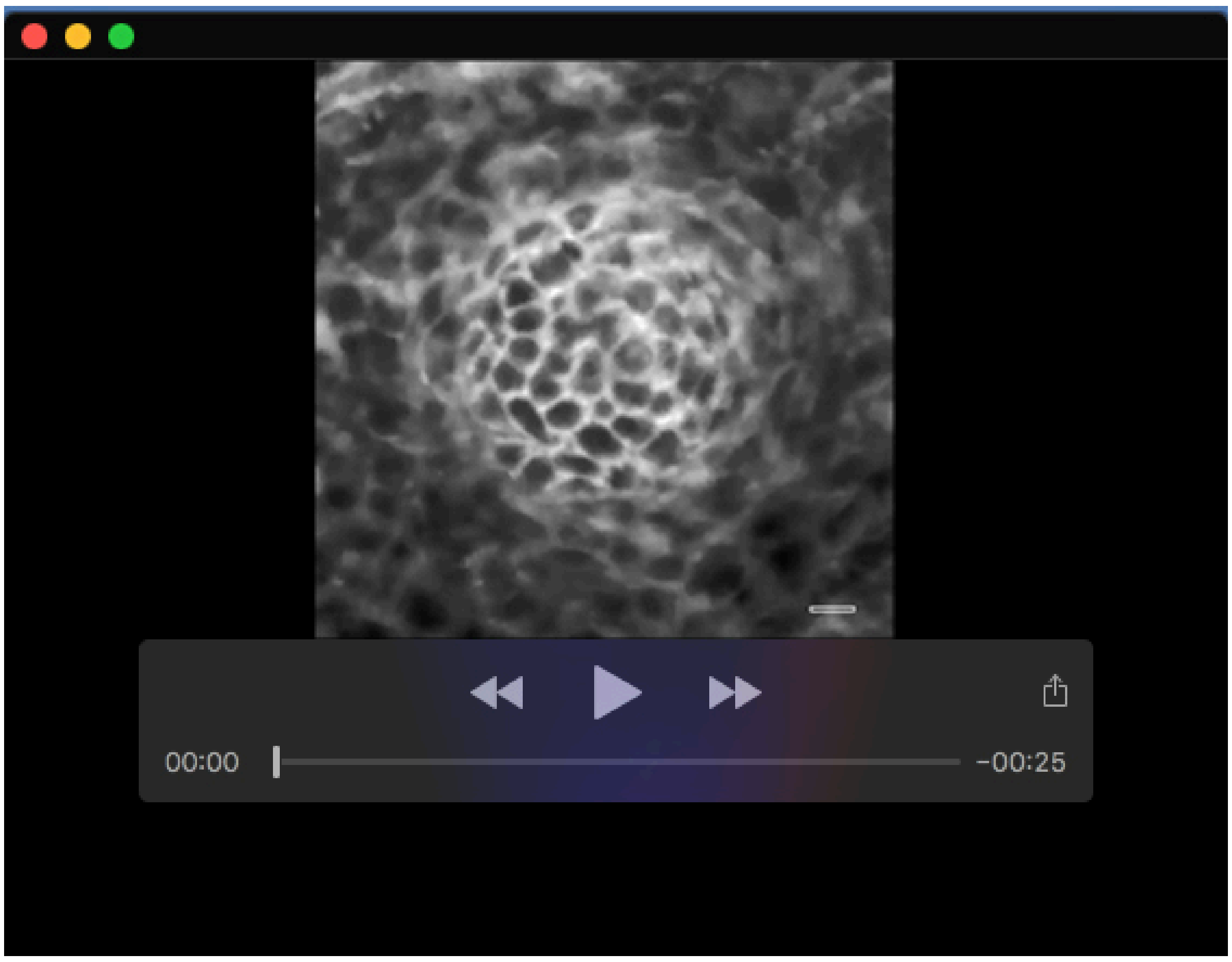

Movie 6. PCP-directed collective cell movements are reversed in posterior *rosette* placodes. Live imaging of an additional posterior *rosette* placode expressing mTomato to label cell membranes. 17 hours/ 20 minute interval. Scale bar, 10 µm. Anterior is to the left.

Development • Supplementary information
